# Physiological Responses and Heat Tolerance Evaluation of Eight Varieties of *Primula vulgaris* Under Natural High Temperatures

**DOI:** 10.3390/plants15071000

**Published:** 2026-03-25

**Authors:** Ruicheng Li, Jiawei Yang, Xin Meng, Chen Cheng, Yingying Zhang, Xueying Han, Nuoxuan Liu, Liyuan Zhao, Ying Qu, Tianqi Tang, Huale Chen, Long Li, Qianqian Shi

**Affiliations:** 1 College of Landscape Architecture and Art, Northwest A&F University, Yangling 712100, China; liruicheng@nwafu.edu.cn (R.L.); yang199972@163.com (J.Y.); 15862639930@nwafu.edu.cn (X.M.); 2024057054@nwafu.edu.cn (C.C.); zhangyingying0316@nwafu.edu.cn (Y.Z.); 2024057013@nwafu.edu.cn (X.H.); lnxx@nwafu.edu.cn (N.L.); zhaoliyuan1011@163.com (L.Z.); 17335570393@163.com (Y.Q.); tangtq@nwafu.edu.cn (T.T.); chenhuale@nwafu.edu.cn (H.C.); 2 Co-Innovation Center for Sustainable Forestry in Southern China, Nanjing Forestry University, Nanjing 210037, China; lilong1949@126.com; 3 Bamboo Research Institute, Nanjing Forestry University, Nanjing 210037, China

**Keywords:** correlation analysis, heat tolerance evaluation, heat-responsive gene expression, natural high temperatures, physiological response, *P. vulgaris*

## Abstract

*Primula vulgaris* possesses considerable edible, medicinal, and ornamental value. It is widely applied in food and pharmaceutical development and, as an early-spring flowering plant, is used in landscaping. However, its range of applications and scope are significantly limited due to its inability to withstand high temperatures. This study aimed to investigate the heat tolerance of *P. vulgaris* under natural high temperatures during summer, identify the most heat-resistant varieties, and determine the optimal conditions for summer outdoor cultivation. Eight *P. vulgaris* varieties were selected and placed under forest shade with three different shading rates during the summer high-temperature period. Additionally, the heat damage index and the following six physiological indicators were measured: malondialdehyde (MDA) content, superoxide dismutase (SOD) activity, peroxidase (POD) activity, soluble sugar content, soluble protein content, and relative conductivity. Furthermore, a correlation analysis of the physiological indicators was conducted, and a heat tolerance evaluation was performed using the membership function method. Simultaneously, qRT-PCR was employed to analyze the expression patterns of three heat stress-related genes (*PvHSP70*, *PvNCED6*, and *PvHSF24*) across the different cultivars and experimental sites. Under heat stress conditions, leaf area was found to be positively and highly significantly correlated with stomatal density (*p* < 0.01). The heat damage index, MDA content, and relative conductivity increased significantly with prolonged stress, and they showed highly significant positive correlations. SOD activity, soluble sugar content, and soluble protein content increased to resist heat damage, while POD activity exhibited no consistent trend. Highly significant positive correlations were observed among protective enzyme activities and osmotic regulatory substances. After a comprehensive evaluation, the eight varieties were ranked according to heat tolerance as follows: “Early Punas Yellow” > “Danova Red” > “Middle Punas Rose Red” > “Middle Punas Blue” > “Middle Punas Red” > “Danova Rose White” > “Middle Punas Crimson” > “Middle Punas Scarlet”. Conclusions: “Early Punas Yellow”, “Danova Red”, and “Middle Punas Rose Red” demonstrated strong heat tolerance. In addition, the expression of *PvHSP70* and *PvHSF24* was significantly upregulated in heat-tolerant cultivars, while that of *PvNCED6* showed a sustained increasing trend with rising temperatures. The results of a three-way ANOVA suggested that *P. vulgaris* exhibited different regulatory patterns among various traits under natural high-temperature stress. Morphological and integrative damage-related indicators, including leaf area, stomatal density, and the heat damage index, all presented significant “site × time” interaction effects. Meanwhile, some physiological regulatory indicators displayed more complex and inconsistent response patterns. These findings further confirm that a dense forest understory grassland is an ideal environment for the summer outdoor cultivation of *P. vulgaris*.

## 1. Introduction

*Primula vulgaris* is a perennial herbaceous plant of the genus *Primula*, and it has considerable medicinal and edible value. Its flowers and leaves are nutritious and have been widely employed in many fields of traditional and modern medicine. It is one of the most widely used species of *Primula* plants and has been widely commercialized and applied around the world [1]. The flowers and leaves of *P. vulgaris* are rich in vitamin C and contain various bioactive compounds. They are typically utilized in salads, soups, and desserts to enhance immune function, prevent colds, and relieve coughs and sore throats. In traditional Chinese medicine, extracts of *Primulas* are widely adopted to treat diseases such as arthritis and rheumatism. Modern pharmacological studies have revealed that they have anti-inflammatory, antioxidant, and anti-diabetic biological effects [2]. As a result of flowering period regulation, *P. vulgaris* can now bloom year-round, providing considerable value in landscaping applications. It is also an excellent ground cover plant because of its bushy foliage and low-growing habit [3]. However, *P. vulgaris* is rarely used in outdoor cultivation because of its poor tolerance to high summer temperatures [4]. It is commonly grown as an annual or biennial plant in commercial production to lower greenhouse maintenance costs. Consequently, the challenge of summer survival significantly limits its range of applications, usage periods, and geographical distribution, contributing to increasing production management costs and difficulties, as well as preventing the plant from achieving its full landscaping potential [5].

Temperature is a crucial factor affecting plant growth and development. High-temperature stress induces physiological disorders in plants, leading to significant alterations in nutrient metabolism, photosynthesis, cell membrane stability, and enzyme activity [6]. In terms of morphological changes, *Cucumis melo* experiences suppressed seedling and root growth at temperatures above 40 °C, and at 36 °C, pollen viability and pollination success significantly decrease [7]. The seeds of *Trifolium repens* fail to germinate under 40 °C stress, and the germination rate decreases as the temperature rises within the range of 20–35 °C [8]. A study conducted in southern Brazil during two consecutive growing seasons found that, when the maximum daily temperature reached 35 °C, irreversible heat damage to the flower buds and flowers of dahlia began to occur [9]. At the microscopic level, leaf structure plays an important role in plant heat tolerance, with the opening and closing of stomata directly influencing photosynthetic efficiency. Stomatal conductance (gs) is an indicator used to assess the degree of stomatal opening and is closely related to photosynthesis efficiency. Research has shown significant differences in the chlorophyll content changes and ultrastructural stability of chloroplasts in different varieties of *Clematis* sp. under high-temperature stress, indicating that the stability of the microscopic structure under heat stress is closely related to its heat tolerance [10]. Under high-temperature stress, the heat tolerance of *P. vulgaris* is significantly influenced by anatomical features such as the tight arrangement of mesophyll cells, leaf thickness, the number of open stomata, and epidermal hair characteristics [11]. In ivy, under high-temperature stress of 35–40 °C, the disruption of the chloroplast membrane structure and the disorganization of cell membrane compartments affect the Hill reaction and photosynthetic function. However, these ultrastructural changes in chloroplasts, in the absence of necrotic damage, are reversible, suggesting that the stability of cellular ultrastructure is closely related to heat tolerance under high-temperature conditions [12].

In recent years, multiple gene families have been demonstrated to play critical roles in regulating plant thermotolerance. Previous studies have revealed that small heat shock protein genes *PHSP21.4* and *PHSP17.1* are essential for high-temperature stress responses in Primula, being not only closely associated with thermotolerance but also involved in drought and salinity tolerance [13,14]. In rice, the key ABA biosynthetic gene *OsNCED1* has been shown to significantly enhance thermotolerance during the heading and flowering stages, as evidenced by improved pollen viability, seed-setting rates, and antioxidant enzyme activities, along with reduced oxidative damage [15]. Heat shock transcription factors (HSFs) represent another central component of thermotolerance regulation, primarily functioning as transcriptional activators that induce the expression of HSPs. Under normal conditions, the interactions between HSFs and molecular chaperones such as Hsp90, Hsp70, and Hsp17 are crucial for plant viability and stability [16].

Currently, the methods for assessing plant heat tolerance primarily include field identification, artificial climate simulation, and the membership function method [17]. Field identification is straightforward and commonly used for crops such as rice, maize, radishes, and various vegetables. However, it is greatly influenced by environmental factors, is time-consuming, and faces challenges in meeting the needs of large-scale, precise screening. The membership function method integrates multiple indicators to avoid the one-sidedness of single evaluation criteria, making it suitable for comprehensive assessments of plant heat tolerance. It has been widely applied to various species of *P. vulgaris* [18,19]. Findings show that the heat damage index, relative conductivity, MDA content, proline, and soluble protein content can serve as important indicators for evaluating the heat tolerance of *P. vulgaris* [20]. Moreover, heat tolerance also involves regulation at the leaf structure and molecular levels [21]. However, most existing studies are based on artificial simulations, overlooking the real physiological impact of natural high-temperature fluctuations on plants [22]. Therefore, this study aims to combine field identification with the membership function method in order to construct a heat tolerance evaluation system that balances accuracy and systematic assessment.

In this study, eight *P. vulgaris* varieties from two series with high ornamental value and broad market applications were selected. The morphological changes and physiological indicators of *P. vulgaris* under different shading rates in shaded environments during the summer’s natural high temperatures were observed, followed by a comprehensive evaluation of heat tolerance. This study preliminarily explored the summer survival conditions of these plants, and it provides a scientific reference for further investigations on the effects of high-temperature stress on plant physiology and morphology.

## 2. Materials and Methods

### 2.1. Plant Materials

The experimental materials were potted *P. vulgaris* seedlings purchased from Jiaxing Shuimu Landscaping Engineering Co., Ltd., Jiaxing, China. Eight varieties from two series with high ornamental value and widespread cultivation application were selected: “Middle Punas Blue” (MPB), “Danova Rose White” (DRW), “Danova Red” (DR), “Middle Punas Crimson” (MPC), “Middle Punas Scarlet” (MPS), “Middle Punas Red” (MPR), “Middle Punas Rose Red” (MPRR), and “Early Punas Yellow” (EPY) (labeled 1–8, respectively). The seedlings were simultaneously sown in pots, exhibited uniform growth, and showed no signs of pests or diseases.

From 12 May to 6 August 2023, the potted seedlings of the eight *P. vulgaris* varieties were transferred from a constant-temperature greenhouse to three different experimental sites. Five pots (15 cm in diameter) of each variety were randomly placed at each site, for a total of 120 pots. The soil in each pot was a mixture of peat soil and perlite in a 3:1 ratio. General-purpose fertilizer (Flower No. 1) was diluted at a ratio of 1:1000 and applied to the soil. The seedlings were placed in nursery pots with a diameter of 15 cm, filled to two-thirds of the pot’s height. Watering was performed every 2–5 days using a watering can, and irrigation was considered sufficient when water droplets appeared at the drainage holes or when the pot noticeably increased in weight.

According to the definition proposed by Jagadish et al., high-temperature stress occurs when the daily maximum temperature exceeds 30 °C for more than two consecutive days, with each day experiencing at least 6 h of such temperatures [23,24,25]. This study began on 12 May, which corresponds to the 8th day after the onset of summer according to the traditional East Asian solar calendar, and ended on 6 August. By that time, the daily maximum temperature at the experimental sites had consistently exceeded 30 °C, placing *P. vulgaris* under high-temperature stress conditions. During the stress period, five mature and healthy leaves from the same positions (the 4th–5th leaves from the bottom) were randomly collected from each variety on 12 May, 9 June, 7 July, and 6 August. To collect the leaf samples at each time point, a destructive sampling method was used rather than performing repeated measurements on the same leaf. After removing the petioles and main veins, the leaves were quickly frozen in liquid nitrogen and stored at −80 °C for physiological measurement and qRT-PCR analysis. Fresh leaves were randomly collected for leaf area and stomatal structure measurements.

### 2.2. Experimental Location

**Figure 1:**
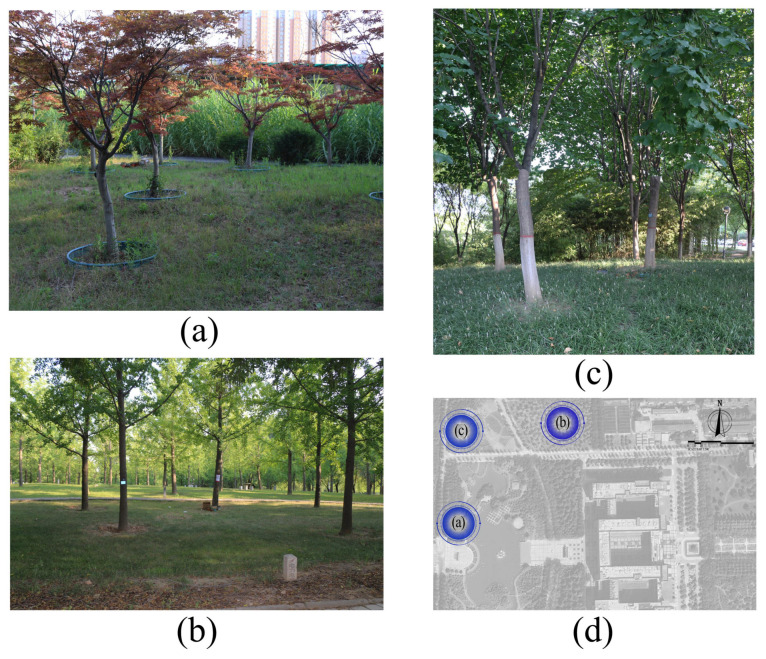
Test site environments: (**a**) Site 1, (**b**) Site 2, and (**c**) Site 3. (**d**) Locations of the experimental sites.

From 12 May to 6 August 2023, the experiment was conducted at three forest-shaded lawn sites on the South Campus of Northwest A&F University. The sites were flat, had the same orientation, and differed in shading rates (Figure 1). During the experimental period, temperature and humidity recorders (RTS-108, Shenzhen Ruitesi Instrument Co., Ltd., Shenzhen, China) were installed at each experimental site to record the daily maximum temperature and relative humidity at 16:00 (Figures S1 and S2). The three sites exhibited consistent trends in the maximum temperature variation, though significant differences were observed: the highest recorded temperature was 40.6 °C at Site 1, followed by 35.9 °C at Site 2 and 33.6 °C at Site 3. The corresponding minimum temperatures were 13.4 °C, 10.0 °C, and 10.2 °C, respectively (Figure S2). The sampling temperatures were based on the average daily maximum temperature over the three days preceding each sampling date. Across the four sampling periods, temperatures at all sites continuously increased. However, with increasing shade, temperatures decreased significantly (Table S2). In terms of relative humidity, the value at Site 1 was the lowest (23%), while that at Sites 2 and 3 reached up to 99% (Figure S2). Additionally, on three sunny, windless days with strong midday sunlight, illuminance was measured at five points located at the canopy center, 1.5 m above the ground, to determine shading rates [26,27], using full-light background levels as a reference (Wang et al., 2013; Chen Sheng, 2022). During the experimental period, the shading rate of Site 1 was 60–70%, that of Site 2 was 91–93%, and that of Site 3 ranged from 89% to 98% (Table S3). A total of 21 rainy days were recorded, primarily concentrated in early June (Figure S3); however, rainfall did not significantly affect the light conditions at the sites.

In summary, Site 1 was located on red maple (*Acer rubrum*) understory grassland and had the highest temperature, lowest humidity, and lowest shading rate (Figure 1a). Site 2 was situated on sparse ginkgo (*Ginkgo biloba*) understory grassland, with intermediate values for all measured variables (Figure 1b). Site 3 was located on dense Chinese redbud (*Cercis chinensis*) understory grassland in Hubei, exhibiting the lowest temperature, highest humidity, and highest shading rate (Figure 1c). During the experiment, ultraviolet index fluctuations were consistent across all sites, and soil temperature showed no significant differences; thus, these variables were excluded from subsequent analyses (Table S1).

### 2.3. Morphological and Physiological Measurements

#### 2.3.1. Leaf Area Reduction Rate

For each *P. vulgaris* cultivar at the three experimental sites, the leaf area measured on 12 May (5–12) was used as the baseline. Subsequent measurements were recorded throughout the experimental period, and the leaf area reduction rate was calculated using Equation (1):

Leaf area reduction rate = (Initial leaf area − Final leaf area)/Initial leaf area × 100%(1)

#### 2.3.2. Stomatal Structure of Leaves

At each of the three experimental sites, five mature, healthy, and pest-free functional leaves were randomly collected from the 4th to 5th fully expanded leaves (from the base) of each tested plant. The abaxial epidermis (~1 × 1 cm^2^) was gently scraped using a scalpel and placed on a clean glass slide. A drop of distilled water was added to maintain moisture, and a temporary mount was prepared by covering with a coverslip. Stomatal observations were conducted using an Olympus BX53 + DP80 microscope (Olympus Corporation, Tokyo, Japan). For each leaf, five replicate slides were prepared, and five random fields of view were observed at 400× magnification. Stomatal density (number per mm^2^) was calculated as the average number of stomata per unit area, following the method described by Royer [28] and Sack and Buckley [29].

#### 2.3.3. Heat Damage Index Observation

Referring to previous studies on heat damage grading for melon seedlings under natural high temperatures, combined with the characteristics of leaf changes and heat damage symptoms in *P. vulgaris* [30], the grading standards for heat damage symptoms were established as follows:

Grade 0: Normal plant growth with no heat damage symptoms.

Grade 1: Good plant growth, with ≤20% of the leaf area showing scorching, wilting, or curling.

Grade 2: Average plant growth, with 20–40% of the leaf area showing scorching, wilting, or curling.

Grade 3: Poor plant growth, with 40–60% of the leaf area showing scorching, wilting, or curling.

Grade 4: Very poor plant growth, with 60–80% of the leaf area showing scorching, wilting, or curling.

Grade 5: The entire plant is dried out, severely dehydrated, and either dead or near death.

Heat Damage Index = Σ(Heat Damage Levels per Plant for Each Variety in Each Experimental Site)/4(2)

where 4 represents the number of sampling periods (12 May, 9 June, 7 July, and 6 August).

#### 2.3.4. Physiological Indicator Measurements

The MDA content was determined using the thiobarbituric acid method. SOD activity was measured using the nitroblue tetrazolium photoreduction method. POD activity was determined using the guaiacol method. The soluble sugar content was measured using the anthrone reagent method. The soluble protein content was determined using the Coomassie Brilliant Blue method. Relative conductivity was measured using the electrical conductivity method [31,32]. Each physiological indicator was measured with five biological replicates and five technical replicates.

### 2.4. Quantitative Real-Time PCR Analysis

To analyze the expression patterns of heat stress-related genes in *P. vulgaris* under high-temperature stress, three genes, *PvHSP70*, *PvNCED6*, and *PvHSF24*, were selected for quantitative real-time PCR (qRT-PCR) analysis. Total RNA was extracted from the leaf samples using a commercial kit (Tiangen Biotech Co., Ltd., Beijing, China), and genomic DNA contamination was removed with DNase I (Takara Bio Inc., Shiga, Japan). Subsequently, 2 μg of total RNA was used to synthesize first-strand cDNA with M-MLV reverse transcriptase (Promega, Madison, WI, USA). qRT-PCR was then performed following the method described by Shi et al. [33], with Actin (c55882.graph_c0) used as the reference gene and the heat-sensitive *P. vulgaris* cultivar MPS used as the calibrator control. Relative gene expression levels were calculated using the 2^−ΔΔCt^ method. Each reaction included three biological replicates and three technical replicates. Gene information was obtained from published transcriptome data (NCBI SRA accession no. SRP120574), and gene-specific primers were designed using Primer3 software (Table S4).

### 2.5. Comprehensive Heat Tolerance Evaluation

Considering that shading may affect the physiological indicators, the data from Site 1, which had the lowest shading rate and the highest temperature, were used for a heat tolerance evaluation and correlation analysis of the indicators. This environmental condition more closely resembles a direct heat stress environment, making it more suitable as a basis for evaluating the intrinsic heat tolerance of different varieties. On this basis, principal component analysis (PCA) combined with the membership function method was used to comprehensively assess the heat tolerance of *P. vulgaris*. In addition, to evaluate the consistency of variety performance under different environmental conditions, the same method was applied to analyze the data from Sites 2 and 3. The results were then compared with those from Site 1 and further verified through a three-factor analysis of variance.

The specific calculation methods were based on previous studies:

Heat Tolerance Coefficient (*Z_ij_*) = Treatment Value/Control Value × 100% (3)

Comprehensive Indicator Score (*C_ik_*) = ∑X_kj_*Z_ij_ (4)

If the heat damage index was positively correlated with heat tolerance, then the following equation was used:

Membership Function Value (*U_ik_*) = (*C_ik_* − *C_kmin_*)/(*C_kmax_* − *C_kmin_*) × 100% (5a)

If the heat damage index was negatively correlated with heat tolerance, then the following equation was used:

Membership Function Value (*U_ik_*) = 1 − (*C_ik_* − *C_kmin_*)/(*C_kmax_* − *C_kmin_*) × 100% (5b)

Comprehensive Indicator Weight (*W_k_*) = *P_k_*/Σ*P_k_* (6)

Comprehensive Heat Tolerance Evaluation Value (*D_i_*) = Σ(U_ik_*W_k_) (7)

In the formulas, the following notations are used:

*Z_ij_* represents the heat tolerance coefficient of the j-th indicator for the i-th variety.

*C_ik_* represents the score of the i-th variety on the k-th principal component.

*X_k_* represents the indicator coefficient of the j-th indicator in the k-th principal component.

*U_ik_* represents the membership function value of the k-th principal component for the i-th variety.

*C_kmax_* and *C_kmin_* represent the maximum and minimum scores of the k-th comprehensive indicator across all varieties.

*W_k_* represents the weight of the k-th comprehensive indicator.

*P_k_* represents the contribution rate of the k-th comprehensive indicator in the principal component analysis.

*D_i_* represents the comprehensive heat tolerance evaluation value of the i-th variety.

### 2.6. Data Statistics and Analysis

Data were processed using ImageJ (version 2023) and Microsoft Office Excel (version 2016). A three-way analysis of variance (ANOVA) and multiple comparison tests were performed using SPSS software (version 26.0). Graphs were generated using Origin (version 2021), GraphPad Prism (version 10). Adobe Photoshop (version 2021) was used only for figure layout and image processing.

## 3. Results

### 3.1. Changes in the Heat Damage Index of P. vulgaris Leaves Under Natural High Temperatures

#### 3.1.1. Leaf Area

**Figure 2:**
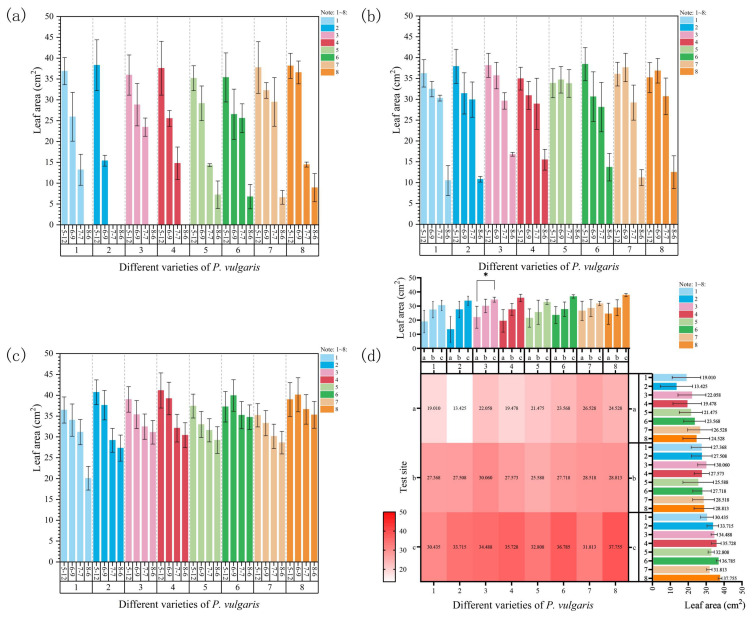
Changes in leaf area of *Primula vulgaris* varieties under natural high temperatures. (**a**) Site 1; (**b**) Site 2; (**c**) Site 3; (**d**) Heatmap and mean comparison across Sites 1–3. Error bars represent the mean ± standard deviation (SD). In (**d**), a–c indicate Sites 1–3, respectively. * Significant. Note: 1~8: Middle Punas Blue (MPB), Danova Rose White (DRW), Danova Red (DR), Middle Punas Crimson (MPC), Middle Punas Scarlet (MPS), Middle Punas Red (MPR), Middle Punas Rose Red (MPRR), and Early Punas Yellow (EPY).

As shown in Figure 2a, at Site 1, with continuous temperature increases, the leaf area of all *P. vulgaris* varieties exhibited varying degrees of decline compared to the baseline measurement (12 May). The leaf area of MPB, DRW, DR, and MPC decreased significantly, and the leaves eventually became severely necrotic, indicating poor heat tolerance. Notably, the leaves of DRW were already completely necrotic by 7 July. On 7 June, when high temperatures were first observed, the leaf areas of MPS, MPR, MPRR, and EPY showed slight decreases but without significant differences. By 7 July, with increasing temperatures, the leaf areas of MPS and EPY significantly declined, and they reached their lowest values on 8 August. In contrast, those of MPR and MPRR showed a sharp drop on 7 July. Overall, the leaf area of DRW was initially the largest, but it declined at the fastest rate. In comparison, EPY maintained a relatively high leaf area throughout the experiment, with only about a 77% reduction by the end of the study, making it the most stable among all varieties in terms of leaf area retention.

At Site 2, on 7 June, the leaf areas of MPS, MPRR, and EPY increased, while those of the remaining varieties declined (Figure 2b). As temperatures continued to rise, by 7 July, all varieties exhibited a downward trend in leaf area. However, due to the relatively lower temperatures at this site, the extent of the decline was not significant. During the later stages of high-temperature stress, the leaf areas of all varieties decreased markedly, reaching their minimum values. Among them, MPS showed poor heat tolerance, with its leaf area ultimately dropping to zero. In contrast, DR showed the slowest decline in leaf area, with a final value equal to 64% of its initial measurement, indicating relatively strong heat tolerance.

At Site 3, during the early stage of heat stress on 7 June, all varieties, except for MPR and EPY, showed a decrease in leaf area (Figure 2c). As temperatures further increased by 7 July, the leaf area of all varieties declined, though not significantly. By 6 August, the end of the heat stress period, no plant mortality was observed, and the minimum leaf area of all varieties remained above 20 mm^2^. These results indicate that EPY had the strongest heat tolerance, with a leaf area reduction of only 9%, whereas MPB showed weaker performance, exhibiting the highest reduction rate at 45%.

A comprehensive comparison across the three experimental sites revealed that all cultivars showed the smallest leaf area at Site 1, whereas they maintained the highest leaf area at Site 3, reflecting the best overall performance (Figure 2d). Among the cultivars, DRW demonstrated the most pronounced difference in leaf area between Sites 1 and 3, indicating a significant site-dependent variation. According to a three-way ANOVA, the leaf area under natural high-temperature stress was significantly influenced by cultivar, experimental site, and time, with clearly different contributions from each factor (Figure S4). The effect of time, which was the most pronounced (F = 161.24, *p* < 0.001), was substantially greater than that of site (F = 96.42, *p* < 0.001) and cultivar (F = 3.24, *p* < 0.01). Moreover, the “site × time” interaction effect was also highly significant (F = 19.50, *p* < 0.001). In contrast, neither the “cultivar × site” nor the “cultivar × time” interaction effect reached statistical significance. To sum up, under high-temperature stress, variation in leaf area is primarily driven by time–environment interactions, while cultivar differences play a relatively limited regulatory role.

#### 3.1.2. Changes in Stomatal Density

**Figure 3:**
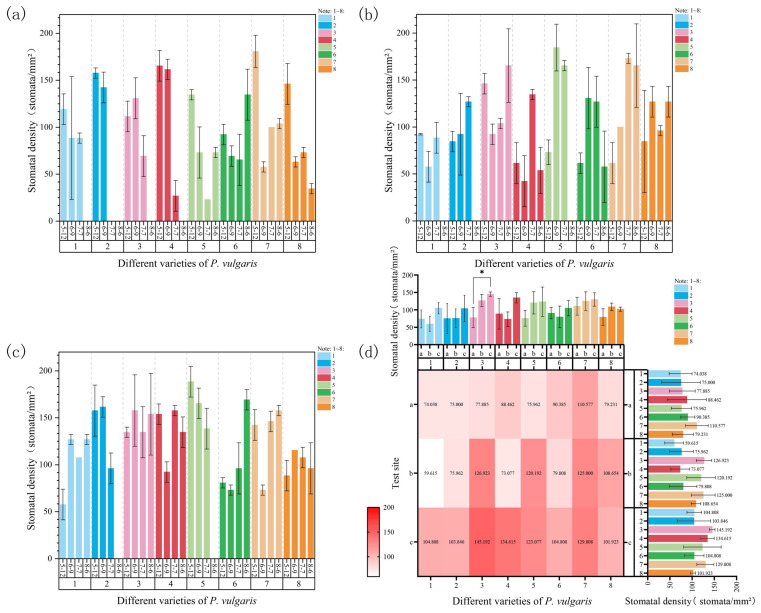
Changes in stomatal density of *P. vulgaris* varieties under natural high temperatures. (**a**) Site 1; (**b**) Site 2; (**c**) Site 3; (**d**) Heatmap and mean comparison across Sites 1–3. Error bars represent the mean ± standard deviation (SD). In (**d**), a–c indicate Sites 1–3, respectively. * Significant. Note: 1~8: Middle Punas Blue (MPB), Danova Rose White (DRW), Danova Red (DR), Middle Punas Crimson (MPC), Middle Punas Scarlet (MPS), Middle Punas Red (MPR), Middle Punas Rose Red (MPRR), and Early Punas Yellow (EPY).

As shown in Figure 3a, at Site 1, during the early stages of high-temperature stress on 7 June, the stomatal density of all cultivars showed a decreasing trend, except for that of DR, which exhibited a slight increase (Figure S5c). As the heat stress intensified, by 7 July, the stomatal density of MPRR and EPY increased, while that of MPB (Figure S5a) and MPR changed only slightly. The other cultivars showed a marked decline. Notably, due to severe leaf dehydration, no observable stomatal structures were detected in DRW on 7 July (Figure S5b). By 6 August, the leaves of four cultivars—MPB, DRW, DR, and MPC—were completely necrotic, and no stomata could be observed. In contrast, MPS, MPR, and MPRR exhibited a significant increase in stomatal density, while EPY showed a marked decline (Figure S5d). As shown in Table S5, under high-temperature stress at Site 1, MPRR exhibited the highest average stomatal density (14.38 ± 3.33), while MPB showed the lowest (9.63 ± 3.34). Meanwhile, DRW exhibited the highest stomatal closure rate, reaching 54%.

As shown in Figure 3b, at Site 2, during the early stages of high-temperature stress on 7 June, DRW (Figure S5b), MPS, MPR, MPRR, and EPY (Figure S5d) exhibited a notable increase in stomatal density, while the remaining cultivars showed a slight decrease. With the further temperature increase on 7 July, all cultivars, except for MPS, MPR, and EPY, which showed slight declines, displayed a marked increase in stomatal density. By 6 August, stomatal structures were no longer observable in the leaves of MPB (Figure S5a), DRW, or MPS. In contrast, DR (Figure S5c) and EPY showed continued increases in stomatal density, while the other cultivars exhibited a decreasing trend. As shown in Table S5, DR had the highest average stomatal density (16.50 ± 2.25), while MPC had the lowest (9.50 ± 2.72). Notably, DRW again exhibited the highest stomatal closure rate at this site, reaching 47%.

As shown in Figure 3c, at Site 3, during the early stages of heat stress (7 June), the stomatal density of MPB, DRW, DR, and EPY showed an increasing trend, while that of the remaining cultivars exhibited varying degrees of decrease. As the stress intensified, by 7 July, the stomatal density of MPB (Figure S5a), DRW (Figure S5b), DR (Figure S5c), MPS, and EPY (Figure S5d) decreased, whereas MPR and MPRR showed a slight increase. By 6 August, with further intensification of heat stress, no observable stomatal structures were found in the leaves of DRW or MPS, while the stomatal density of MPB, DR, MPR, and MPRR increased again; the remaining cultivars showed a slight decline. As shown in Table S5, at Site 3, DR exhibited the highest average stomatal density (18.88 ± 0.80), whereas DRW exhibited the lowest (13.50 ± 4.90), along with the highest stomatal closure rate (32%).

A comparison of stomatal density across the three experimental sites revealed that the response patterns varied among cultivars, with only DR presenting a significant difference in stomatal density between Sites 1 and 3 (Figure 3d). Furthermore, a three-way ANOVA of the data collected in June, July, and August suggested that stomatal density under natural high-temperature stress was significantly affected by experimental site and time, and that the effects of cultivar and most interaction terms were relatively weak (Figure S4). The effect of both site (F = 5.22, *p* < 0.01) and time (F = 4.38, *p* < 0.01) was significant, indicating that stomatal density is more sensitive to environmental conditions under high-temperature stress. Furthermore, the “site × time” interaction effect also reached significance (F = 3.25, *p* < 0.05), whereas neither the “cultivar × site” nor the “cultivar × time” interaction effect was statistically significant. These results suggest that, under prolonged stress, the variation in stomatal density was predominantly governed by the interaction of time and environment, and that the effect of genotypic differences was not statistically significant.

#### 3.1.3. Heat Damage Index

**Figure 4:**
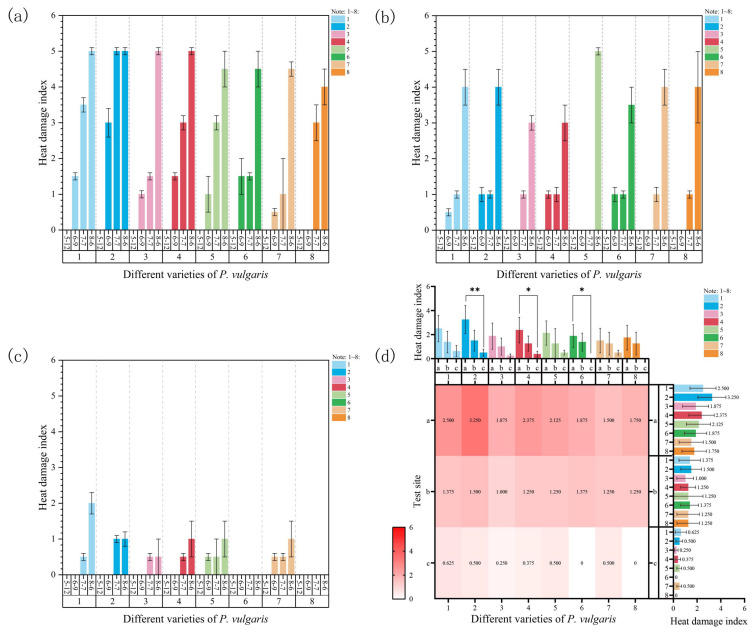
Changes in heat damage index of *P. vulgaris* varieties under natural high temperatures. (**a**) Site 1; (**b**) Site 2; (**c**) Site 3; (**d**) Heatmap and mean comparison across Sites 1–3. Error bars represent the mean ± standard deviation (SD). In (**d**), a–c indicate Sites 1–3, respectively. * Significant; ** highly significant. Note: 1~8: Middle Punas Blue (MPB), Danova Rose White (DRW), Danova Red (DR), Middle Punas Crimson (MPC), Middle Punas Scarlet (MPS), Middle Punas Red (MPR), Middle Punas Rose Red (MPRR), and Early Punas Yellow (EPY).

At Site 1, the heat damage index of all varieties exhibited an overall upward trend as temperatures and the duration of high-temperature exposure increased (Figure 4a). In MPB (Figure S6a), DR (Figure S6c), MPC, and MPS, significant differences in the heat damage index were observed between sampling times, accompanied by progressively expanding scorched and yellowed leaf areas. Eventually, all of the plants dried out and wilted. All DRW plants had died by the time of the sampling procedure on 7 July, with a heat damage index of 5. The heat damage index of this variety remarkably rose during the first three samplings (Figure S6b). The heat damage index of MPR showed no changes during the second and third samplings because of the relatively small temperature differences in these periods; however, by the fourth sampling, it increased significantly when the temperature rose to 39 °C. The heat damage index of MPRR showed no significant changes during the first three samplings but significantly rose during the fourth sampling. Regarding EPY, no noticeable morphological changes were observed before 7 July. After this date, its heat damage index notably increased as temperatures rose (Figure S6d). By the end of the experiment, the heat damage index of MPB, DRW, DR, and MPC reached a maximum value of 5. The plants that had died before or during sampling because of high temperatures were assigned a heat damage index of 5. During the experiment, MPRR had the lowest average heat damage index (1.5). By the third sampling on 7 July, this variety exhibited only mild heat damage, with a few leaves scorched and curled. By 6 August, most leaves had dried out and fallen off due to excessively high temperatures, although some remained healthy. In contrast, DRW exhibited the highest average heat damage index (3.25). By the third sampling on 7 July, this variety had completely dried out, become severely dehydrated, and died.

At Site 2, the heat damage index of all varieties exhibited an overall upward trend (Figure 4b). By the fourth sampling on 6 August, the temperature significantly increased to 34 °C, causing the heat damage index of all varieties to increase and reach maximum values. During the second and third samplings, the temperatures were relatively similar, and the heat damage index showed no significant changes in MPB (Figure S6a), DRW, MPC, MPS, or MPR. However, the heat damage index of DR, MPRR, and EPY notably increased during the third sampling (Figure S6d) despite the absence of a significant temperature change. This might have been caused by the cumulative temperature effect. Under heat stress, DR (Figure S6c) had the lowest average heat damage index (1). By the third sampling, only a very small area on the leaf edges displayed scorching and yellowing. By the fourth sampling, nearly half of the leaf area had turned yellow and dried out. In contrast, DRW showed the highest average heat damage index (1.5). Minor scorching was observed on the leaf edges by the second sampling on 9 June. Moreover, most leaves were curled, dried, or had even fallen off by the fourth sampling (Figure S6b).

During the experiment, the maximum temperature at Site 3 rose to 32 °C. During the first three samplings, the temperature did not exceed 30 °C. Therefore, no heat damage was observed in MPR or EPY throughout the experiment (Figure 4c). MPB, DRW (Figure S6b), DR (Figure S6c), and MPC started showing heat damage on 7 July when the temperature significantly increased and reached a cumulative threshold. MPS and MPRR showed minor heat damage during the second sampling on 9 June, though these changes were not statistically significant. Overall, the varieties at Site 3 experienced relatively mild heat damage, with no significant external morphological changes observed in most plants. MPB recorded the highest average heat damage index (0.625). No heat damage or only very minor symptoms were observed during the first three samplings, while slight yellowing and dehydration were observed in small areas of the leaves by the fourth sampling (Figure S6a). In contrast, MPR and EPY (Figure S6d) had the lowest average heat damage index at 0, showing no visible symptoms of heat damage throughout the experiment.

A comprehensive comparison of the heat damage index among the eight cultivars across the three experimental sites reflected a clear decreasing trend from Site 1 to Site 3 for all cultivars. Notably, the heat damage index of DRW at Site 1 was significantly higher than that at Site 3 (Figure 4d), indicating that the experimental site had a substantial influence on the summer survival of *P. vulgaris*. Furthermore, a three-way ANOVA revealed that the heat damage index under natural high-temperature stress was significantly affected by the combined effects of site and time, whereas the contributions of cultivar and its associated interaction terms were relatively weak (Figure S4). Among all sources of variation, the effect of time was the most pronounced (F = 91.66, *p* < 0.001), followed by that of site (F = 60.10, *p* < 0.001). The “site × time” interaction effect also reached a highly significant level (F = 13.78, *p* < 0.001); however, neither the “cultivar × site” nor the “cultivar × time” interaction effect was significant. Thus, it can be concluded that the heat damage index is primarily regulated by the duration of high-temperature stress, the interaction between environmental conditions, and the “site × cultivar” effect, with the relative strength of these effects following the order of period > site > site × cultivar.

### 3.2. Effects of Natural High Temperatures on Malondialdehyde (MDA) Content of P. vulgaris

**Figure 5:**
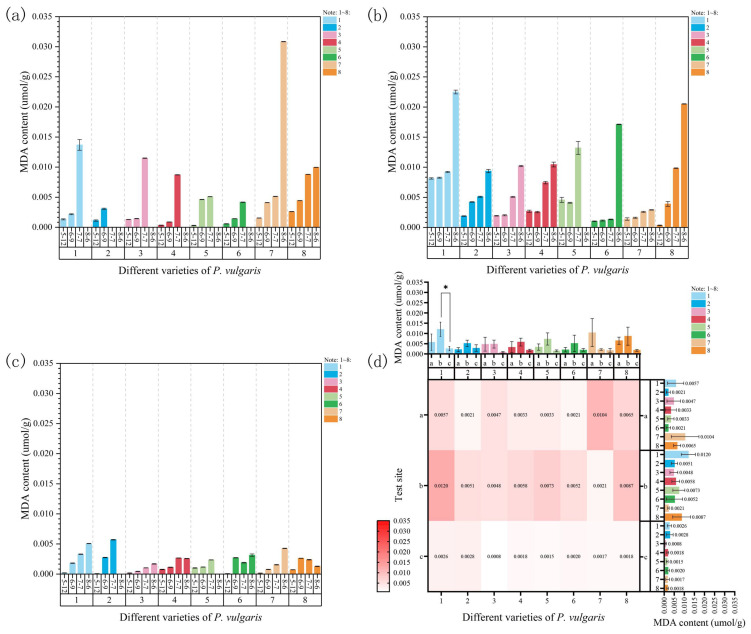
Changes in MDA content in *P. vulgaris* varieties under natural high temperatures. (**a**) Site 1; (**b**) Site 2; (**c**) Site 3; (**d**) Heatmap and mean comparison across Sites 1–3. Error bars represent the mean ± standard deviation (SD). In (**d**), a–c indicate Sites 1–3, respectively. * Significant. Note: 1~8: Middle Punas Blue (MPB), Danova Rose White (DRW), Danova Red (DR), Middle Punas Crimson (MPC), Middle Punas Scarlet (MPS), Middle Punas Red (MPR), Middle Punas Rose Red (MPRR), and Early Punas Yellow (EPY).

At Site 1, the MDA content of all varieties consistently increased with a significant rise in both the sampling temperatures and the duration of stress (Figure 5a). By 7 July, all DRW plants had died under excessively high temperatures. By 6 August, all MPB, DR, MPC, MPR, and MPS plants had also died when the sampling temperature reached 39.87 °C. Concerning the remaining varieties, the MDA content reached its maximum value during the fourth sampling. Significant differences in the MDA content were observed across all sampling times for each variety. Compared to the control group, the increase in the MDA content at 39 °C varied significantly among the varieties. MPRR showed the largest increase (2612.58%), while DRW showed the smallest (223.13%).

At Site 2, the MDA content of all varieties exhibited an overall upward trend with increasing temperatures (Figure 5b). All MPS plants had died by the sampling procedure on 6 August because of excessively high temperatures. In all other varieties, except for MPRR, the MDA content significantly increased with rising temperatures. Compared to the control group, the increase in the MDA content considerably varied among the varieties at 34 °C. EPY exhibited the greatest increase (5796.74%), whereas MPRR showed the smallest (108.53%).

From 7 July to 6 August, the excessively high shading rate at Site 3 resulted in increased air humidity, coupled with continuous rainfall and rising temperatures. Thus, the likelihood of pest and disease outbreaks significantly rose. Consequently, DRW and MPS were completely infected and died before the fourth sampling. The MDA content of MPB, DRW, DR, MPS, and MPRR showed a continuous and significant increase. In MPC, the MDA content dramatically increased during the first three samplings but decreased as the temperature rose on 6 August. The MDA content of MPR slightly decreased on 7 July but demonstrated a fluctuating upward trend overall, reaching its maximum value on 6 August. Notably, EPY experienced a sharp rise in MDA content, peaking on 9 June, followed by a gradual decline thereafter (Figure 5c). By the end of the experiment, DRW showed the highest increase in MDA content (7811.31%), while EPY had the lowest (79.18%).

A comprehensive comparison of the MDA content of the different cultivars across the three experimental sites showed that, except for MPRR, which exhibited the highest MDA content at Site 1, the other eight *P. vulgaris* cultivars displayed the highest MDA contents at Site 2 and the lowest at Site 3 (Figure 5d). The MPB cultivar exhibited a significant difference in MDA content between Sites 2 and 3 (Figure 5d). Additionally, a three-way ANOVA was conducted on the MDA content in June, July, and August. The results suggest that, under natural high-temperature stress, MDA was significantly influenced by both site and temporal effects, whereas the effects of cultivar and all interaction terms were not significant (Figure S4). The effects of both site (F = 8.16, *p* < 0.01) and time (F = 6.76, *p* < 0.001) were significant, while the “site × time” interaction effect was not significant (F = 1.70). In other words, MDA responses to high-temperature stress are primarily governed by the independent influences of environmental conditions and stress duration, with differences among sites not further amplified over time.

### 3.3. Effects of Natural High Temperatures on Protective Enzyme Activity of P. vulgaris

#### 3.3.1. Superoxide Dismutase (SOD) Activity Analysis

**Figure 6:**
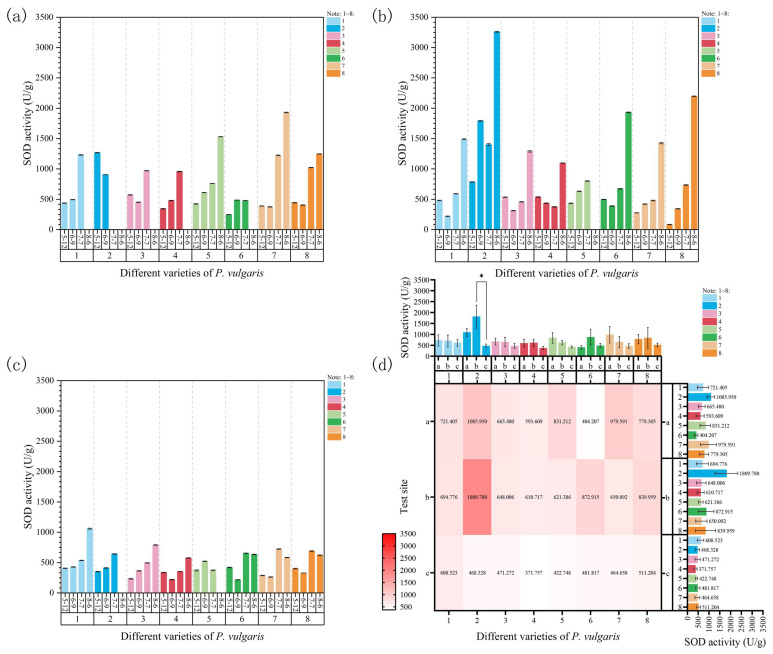
Changes in SOD activity of *P. vulgaris* varieties under natural high temperatures. (**a**) Site 1; (**b**) Site 2; (**c**) Site 3; (**d**) Heatmap and mean comparison across Sites 1–3. Error bars represent the mean ± standard deviation (SD). In (**d**), a–c indicate Sites 1–3, respectively. * Significant. Note: 1~8: Middle Punas Blue (MPB), Danova Rose White (DRW), Danova Red (DR), Middle Punas Crimson (MPC), Middle Punas Scarlet (MPS), Middle Punas Red (MPR), Middle Punas Rose Red (MPRR), and Early Punas Yellow (EPY).

During high-temperature stress at Site 1, the SOD activity of MPB, DR, MPC, and MPS significantly increased with the rising temperatures (Figure 6a). In contrast, MPRR and EPY initially exhibited a decrease in SOD activity at the onset of stress (9 June), followed by a notable increase during the third and fourth samplings. Regarding DRW, a significant reduction in SOD activity was observed under high-temperature stress on 9 June, leading to the death of all plants. Similarly, MPR demonstrated a significant rise in SOD activity during the initial stress period but a sharp decline on 7 July. All plants perished before the fourth sampling, which was attributed to the prolonged exposure to excessive temperatures. Compared to the control group after high-temperature stress, MPRR exhibited the largest increase in SOD activity (394.89%), while DRW showed the greatest decrease (−28.57%), based on the last measurement taken before its death.

At Site 2, the SOD activity of all varieties rose with increasing temperatures (Figure 6b) and reached the maximum values by the end of the experiment. The SOD activity of MPS, MPRR, and EPY significantly increased as the temperature rose. In MPB, DR, and MPR, SOD activity considerably decreased compared to the control group at the onset of stress (9 June). However, their SOD activity subsequently increased, reflecting significant differences. DRW demonstrated a fluctuating upward trend in SOD activity throughout the stress period. In contrast, no significant increase in SOD activity was observed for MPC during the first three samplings, even with a slight decrease. By 6 August, a substantial rise in SOD activity was recorded as the temperature further increased. By the end of the experiment, EPY exhibited the largest increase in SOD activity (2534.72%), whereas MPS showed the smallest increase (84.50%).

At Site 3, the SOD activity of MPB, DRW, and DR significantly increased throughout the experiment (Figure 6c). The SOD activity of MPC decreased significantly compared to that of the control group at the onset of stress on 9 June; however, it subsequently increased as shading and rising temperatures continued, reflecting significant differences. The SOD activity of MPS considerably increased on 9 June but subsequently decreased because of weak light stress induced by continuous shading. By the fourth sampling, all plants had died from disease. In the cases of MPR, MPRR, and EPY, SOD activity initially declined, then increased, and subsequently decreased again, although the overall trend remained upward. Compared to the control group, DR showed the greatest increase in SOD activity (236.53%), while MPS presented the smallest increase (0.95%).

A comparison of SOD activity across the three trial sites revealed that Site 3 exhibited the lowest SOD activity, while no significant difference was observed between Sites 1 and 2 (Figure 6d). Furthermore, a three-way ANOVA indicated that, under natural high-temperature stress, SOD activity was significantly affected by both site and time (Figure S4). Specifically, the site (F = 5.94, *p* < 0.01), time (F = 5.71, *p* < 0.01), and “site × time” interaction effects (F = 3.44, *p* < 0.01) were all significant, with the strength of the main effects being stronger than that of the interaction effects. In other words, the changes in SOD activity were primarily governed by the combined effects of environmental conditions and stress duration. Meanwhile, under different environmental conditions, SOD activity presented significant differences as the stress duration increased. In contrast, the cultivar effect (F = 1.17) and the “cultivar × site” and “cultivar × time” interaction effects were not significant, indicating a relatively limited role of genotype in regulating SOD activity.

#### 3.3.2. Peroxidase (POD) Activity Analysis

**Figure 7:**
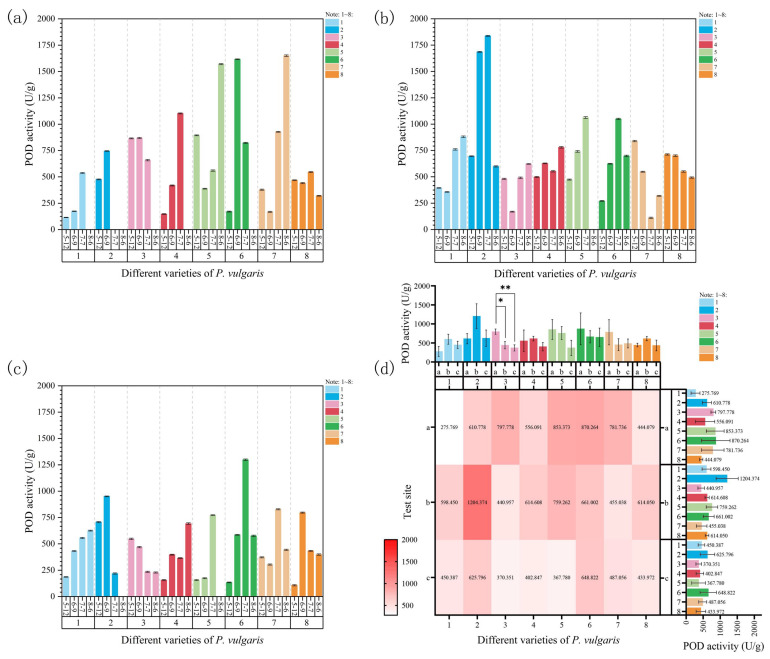
Changes in POD activity of *P. vulgaris* varieties under natural high temperatures. (**a**) Site 1; (**b**) Site 2; (**c**) Site 3; (**d**) Heatmap and mean comparison across Sites 1–3. Error bars represent the mean ± standard deviation (SD). In (**d**), a–c indicate Sites 1–3, respectively. * Significant; ** highly significant. Note: 1~8: Middle Punas Blue (MPB), Danova Rose White (DRW), Danova Red (DR), Middle Punas Crimson (MPC), Middle Punas Scarlet (MPS), Middle Punas Red (MPR), Middle Punas Rose Red (MPRR), and Early Punas Yellow (EPY).

As shown in Figure 7a, the POD activity of MPB, DRW, and MPC continuously increased with rising temperatures and prolonged heat stress, indicating significant differences at Site 1. For DR, no significant changes were observed in POD activity during the first two samplings. However, it decreased significantly on 7 July as the temperature increased further, and, by 6 August, all plants had died due to excessive temperatures. MPS and MPRR presented a significant decrease in POD activity during the initial stages of stress on 9 June. Subsequently, their POD activity increased significantly as the temperature continuously rose, reaching maximum levels by 6 August. MPR exhibited a sharp increase in POD activity in response to high-temperature stress on 9 June. As the temperature continued to rise and the duration of exposure increased, POD activity decreased significantly, and the plants died by 6 August, which was ascribed to extreme heat. In EPY, POD activity displayed a fluctuating upward trend during the first three samplings but decreased significantly by 6 August as temperatures rose. Under high-temperature stress, the greatest increase in POD activity was observed in MPC (648.98%), while EPY showed the largest decrease (−31.40%) compared to the control group.

At Site 2, the POD activity of MPS consistently increased throughout the experiment, presenting significant differences (Figure 7b). The POD activity of MPB, DR, and MPC exhibited a fluctuating upward trend and reached the maximum values under the highest stress temperature on 6 August. In DRW and MPR, POD activity continuously increased during the first three samplings, peaking on 7 July, but decreased as the temperature rose further by 6 August. After the onset of stress, the POD activity of MPRR considerably decreased, but it sharply increased during the fourth sampling as the temperature rose further. In contrast, EPY demonstrated a continuous decline in POD activity throughout the experiment. By the end of the experiment, MPR exhibited the highest increase in POD activity (157.73%), whereas MPRR showed the greatest decrease (−61.82%).

The POD activity of MPB and MPS continuously increased, while that of MPC exhibited a fluctuating upward trend, with all three varieties reaching their maximum POD activity on 6 August at Site 3 (Figure 7c). The POD activity of DR continuously decreased throughout the experiment. In DRW, POD activity increased significantly on 9 June and then sharply declined by the third sampling on 7 July, which was attributed to excessive shading or disease. By 6 August, all plants of this variety had died from disease. The POD activity of MPR and MPRR showed an upward trend after the start of the experiment, peaking on 7 July. However, the plants were damaged as temperatures rose further and excessive shading persisted, leading to a decline in POD activity by 6 August. In EPY, POD activity increased significantly at the start of the experiment (9 June) but decreased continuously and significantly as the experiment progressed. Additionally, MPS recorded the largest overall increase in POD activity (393.33%), while DRW displayed the most significant decrease (−69.01%) compared to the initial control group.

A comparison of POD activity across the three experimental sites revealed that Site 3 had the lowest POD activity levels (Figure 7d). The POD activity of DR at Site 1 was significantly and highly significantly different from that at Sites 2 and 3, respectively (Figure 7d); this indicates that different experimental sites exerted a highly significant impact on the POD activity of Danno Red. Furthermore, a three-way ANOVA of POD activity suggested that this indicator was significantly affected by experimental site and time under natural high-temperature stress (Figure S4). The effects of both site (F = 4.24, *p* < 0.05) and time (F = 4.34, *p* < 0.01) were significant, while those of cultivar (F = 0.70) and all interactions were not significant. To sum up, changes in POD activity are primarily influenced by environmental effects and stress duration, and the regulatory effects of genotype and all interactions are relatively limited.

### 3.4. Effects of Natural High Temperatures on Osmoregulation Substances in P. vulgaris

#### 3.4.1. Analysis of Soluble Sugar Content Changes

**Figure 8:**
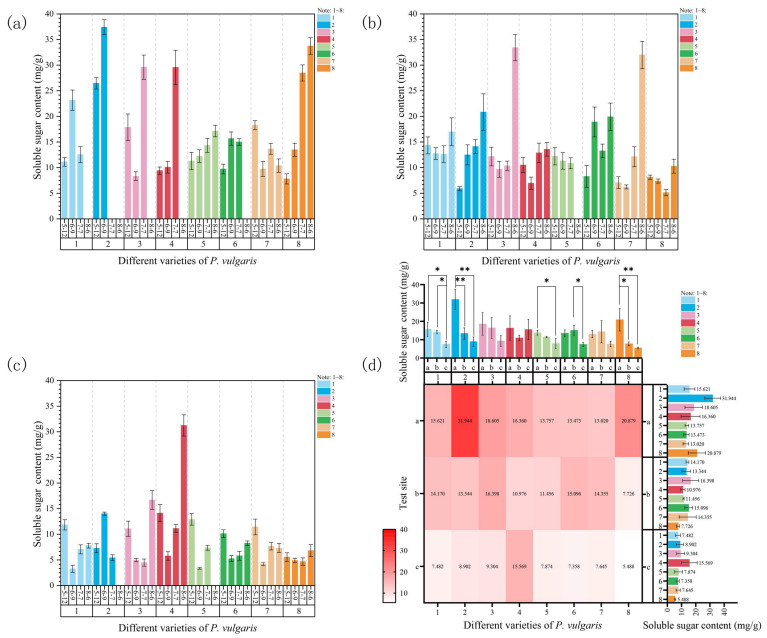
Changes in soluble sugar content of *P. vulgaris* varieties under natural high temperatures. (**a**) Site 1; (**b**) Site 2; (**c**) Site 3; (**d**) Heatmap and mean comparison across Sites 1–3. Error bars represent the mean ± standard deviation (SD). In (**d**), a–c indicate Sites 1–3, respectively. * Significant; ** highly significant. Note: 1~8: Middle Punas Blue (MPB), Danova Rose White (DRW), Danova Red (DR), Middle Punas Crimson (MPC), Middle Punas Scarlet (MPS), Middle Punas Red (MPR), Middle Punas Rose Red (MPRR), and Early Punas Yellow (EPY).

At Site 1, the soluble sugar content of DRW, MPC, MPS, and EPY continuously increased with the rising temperature, as shown in Figure 8a. Except for MPC, which demonstrated no significant increase during the first two samplings, and MPS, which exhibited no significant increase during the first three samplings, the soluble sugar content of all other varieties showed significant differences between sampling times. The soluble sugar content of MPB and MPR significantly increased during the initial stages of high-temperature stress on 9 June and decreased as the stress temperature rose further. By 6 August, all plants of these varieties had died. MPRR presented a fluctuating downward trend in soluble sugar content. In DR, the soluble sugar content decreased at the beginning of the stress period compared to the control group but considerably increased as the temperature rose further. Under high-temperature stress, EPY exhibited the greatest increase in soluble sugar content (332.58%), while MPRR showed the largest decrease (−43.24%) compared to the initial control group.

At Site 2, the soluble sugar content of DRW and MPRR presented an upward trend, with a significant increase and the maximum value reached during the fourth sampling when the temperature rose the most, as illustrated in Figure 8b. Attributed to the relatively small temperature variation during the first three samplings, the soluble sugar content of MPB and DR did not change significantly until the fourth sampling. During the stress period, the soluble sugar content of MPS exhibited a continuous downward trend but without significant differences. In MPC, MPR, and EPY, the soluble sugar content fluctuated but exhibited an upward trend overall as the temperature increased, with all reaching their maximum values on 6 August when the temperature was the highest. By the end of the experiment, MPRR demonstrated the highest increase in soluble sugar content (353.19%), whereas MPS showed the greatest decrease (−11.08%).

At Site 3, the soluble sugar content of all varieties, except for DRW, exhibited a decreasing-then-increasing trend (Figure 8c). After the onset of stress, the soluble sugar content of MPB, MPC, MPS, MPR, and MPRR significantly decreased, reaching the lowest levels on 9 June, and it increased thereafter. Nevertheless, the final levels of all other varieties were significantly lower than those of the control group, except for MPC. In DR and EPY, the soluble sugar contents continuously dropped, reaching their lowest levels on 7 July; however, they remarkably increased during the highest temperature on 6 August, reaching their maximum values. DRW demonstrated a significant increase in soluble sugar content on 9 June, reaching its peak value, followed by a sharp and significant decrease. By 6 August, all plants of this variety had died from disease. Similarly, MPC showed the largest increase in soluble sugar content (121.15%), while MPS again exhibited the most significant decrease (−43.02%) compared to the control group.

An analysis of the soluble sugar content at the three experimental sites indicated that, for each cultivar, it was the highest at Site 1 and the lowest at Site 3, demonstrating a significant decreasing trend with significant differences (Figure 8d). DRW and EPY exhibited highly significant and significant differences in soluble sugar content between Sites 1 and 2, respectively. MPB and MPR displayed significant differences in soluble sugar content between Sites 2 and 3. Many cultivars showed significant differences in soluble sugar content between Sites 1 and 3. Among them, MPB and MPS demonstrated significant differences, whereas DRW and EPY showed highly significant differences (Figure 8d). In other words, the experimental site exerted a significant regulatory effect on the soluble sugar content of the plants. Furthermore, the results of a three-way ANOVA revealed that soluble sugar content under natural high-temperature stress was primarily significantly affected by the experimental site (Figure S4). The site effect was significant (F = 4.52, *p* < 0.05), while the time (F = 0.10) and cultivar effects (F = 0.27) were not significant. Simultaneously, the “site × time” interaction effect was also significant (F = 2.49, *p* < 0.05); in contrast, the interactions between cultivar and site and between cultivar and time were not significant. In conclusion, changes in the soluble sugar content are essentially driven by environmental effects and, to some extent, regulated by time effects; the effect of genotype was not statistically significant.

#### 3.4.2. Analysis of Soluble Protein Content Changes

**Figure 9:**
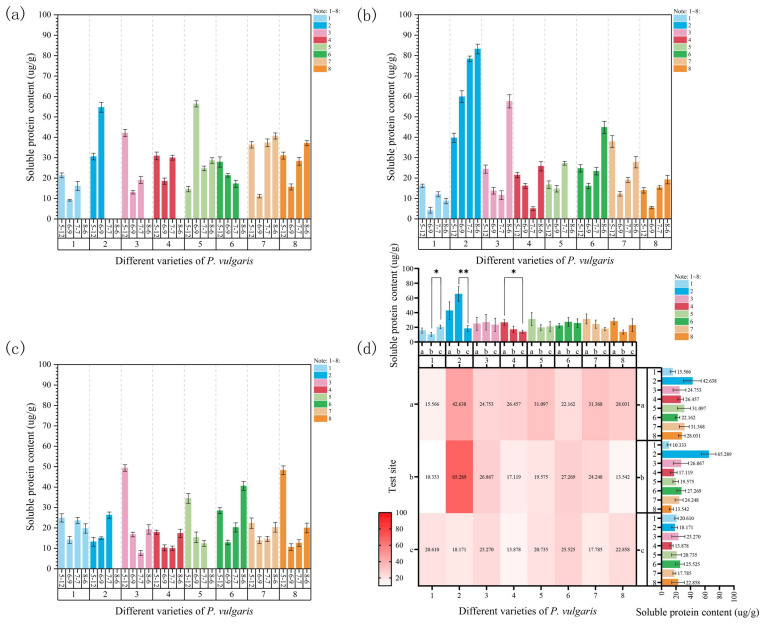
Changes in soluble protein content of *P. vulgaris* varieties under natural high temperatures. (**a**) Site 1; (**b**) Site 2; (**c**) Site 3; (**d**) Heatmap and mean comparison across Sites 1–3. Error bars represent the mean ± standard deviation (SD). In (**d**), a–c indicate Sites 1–3, respectively. * Significant; ** highly significant. Note: 1~8: Middle Punas Blue (MPB), Danova Rose White (DRW), Danova Red (DR), Middle Punas Crimson (MPC), Middle Punas Scarlet (MPS), Middle Punas Red (MPR), Middle Punas Rose Red (MPRR), and Early Punas Yellow (EPY).

At Site 1, the soluble protein content of MPB, DR, MPC, MPRR, and EPY followed a decreasing-then-increasing trend, as shown in Figure 9a. At the onset of stress, the soluble protein content significantly decreased but rose as the temperature increased. In DRW, the soluble protein content notably increased at the beginning of the stress period. However, all plants died during the third and fourth samplings, owing to excessively high temperatures. The soluble protein content of MPS increased significantly on 9 June, reaching its maximum value, and then it dropped sharply. Nevertheless, the final soluble protein content of this variety was considerably higher than that of the control group. In contrast, MPR showed a continuous and significant decline in soluble protein content as the temperature rose, and all plants died by 6 August because of excessive heat. Under high-temperature stress, MPS showed the greatest increase in soluble protein content (95.59%), while DR exhibited the largest decrease (−54.76%) compared to the initial control group.

At Site 2, the soluble protein content of all varieties, except for DRW, followed a decreasing-then-increasing trend (Figure 9b). In MPS, MPR, MPRR, and EPY, the soluble protein content decreased at the onset of stress on 9 June, reaching its lowest levels, with all but MPS showing significant differences. Their soluble protein content increased significantly as the temperature continued to rise and high-temperature stress persisted. The soluble protein content of MPB dropped significantly to its lowest level on 9 June, followed by a fluctuating upward trend. In DR and MPC, the soluble protein contents continuously decreased from the start of the stress period, reaching their lowest levels on 7 July; however, they rose sharply as the temperature significantly increased by the fourth sampling on 6 August. DRW, in contrast, exhibited a consistently significant upward trend in soluble protein content throughout the experiment as the temperature increased (Figure 9b). By the end of the experiment, DR demonstrated the highest increase in soluble protein content (136.35%), whereas MPB recorded the greatest decrease (−45.92%).

At Site 3, the soluble protein contents of MPR, MPRR, and EPY significantly decreased at the beginning of the experiment, reaching their lowest levels on 9 June, as shown in Figure 9c, and, subsequently, they considerably increased. In DR and MPC, the soluble protein contents continuously declined, reaching their lowest levels on 7 July; however, they dramatically increased as the temperature rose further by the fourth sampling. During the experiment, the soluble protein content of DRW and MPS consistently increased and decreased, respectively. In MPB, the soluble protein content showed a fluctuating downward trend, eventually becoming significantly lower than that of the initial control group. At the end of the experiment, the soluble protein content of DRW increased the most, by 98.96% (compared with the last measured data before death), while that of MPS decreased the most, by −64.07%.

A comparative analysis across the three experimental sites revealed that soluble protein contents were generally lower at Site 3 (Figure 9d). Specifically, MPC presented a significant difference between Sites 1 and 3; MPB and DRW exhibited significant and highly significant differences, respectively, between Sites 2 and 3. This further indicates that the environmental conditions at Site 3 exerted a significant inhibitory effect on protein accumulation in different genotypes. Furthermore, a three-way ANOVA of the soluble protein content in June, July, and August suggested that this indicator was significantly affected by cultivar and the “site × time” interaction under natural high-temperature stress (Figure S4). The cultivar effect was significant (F = 2.35, *p* < 0.05), while the site (F = 1.61) and time effects (F = 2.27) were not significant. Simultaneously, the “site × time” interaction effect was significant (F = 3.39, *p* < 0.01), while the effects of other interactions were not significant. To sum up, under high-temperature stress conditions, the changes in soluble protein content were regulated by both cultivar and site × time interaction effects.

### 3.5. Effects of Natural High Temperatures on Relative Conductivity of P. vulgaris

**Figure 10:**
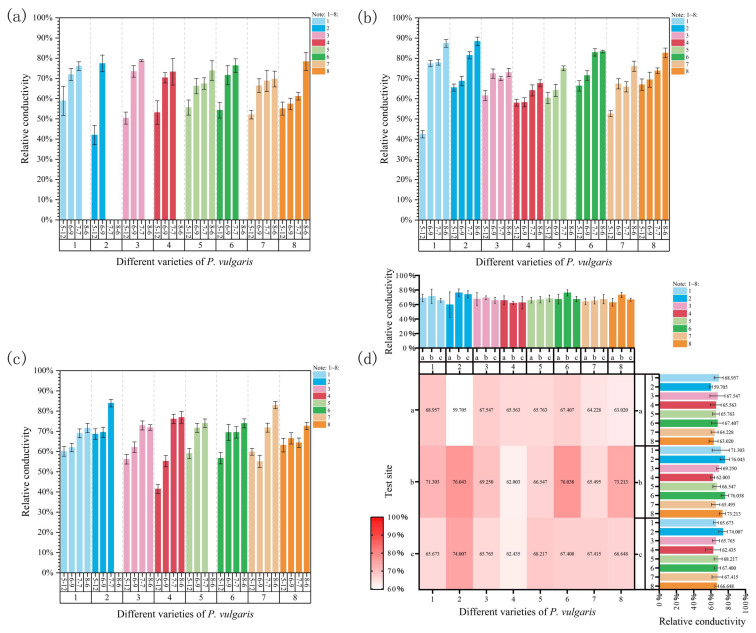
Changes in relative conductivity of *P. vulgaris* varieties under natural high temperatures. (**a**) Site 1; (**b**) Site 2; (**c**) Site 3; (**d**) Heatmap and mean comparison across Sites 1–3. Error bars represent the mean ± standard deviation (SD). In (**d**), a–c indicate Sites 1–3, respectively. Note: 1~8: Middle Punas Blue (MPB), Danova Rose White (DRW), Danova Red (DR), Middle Punas Crimson (MPC), Middle Punas Scarlet (MPS), Middle Punas Red (MPR), Middle Punas Rose Red (MPRR), and Early Punas Yellow (EPY).

During high-temperature stress, the relative conductivity of all varieties at Site 1 continuously increased with rising temperatures and prolonged heat stress (Figure 10a). The relative conductivity of MPB, DRW, MPR, and EPY significantly increased throughout the experiment. In DR, MPC, and MPRR, the relative conductivity considerably increased compared to the control group at the onset of stress; however, subsequent increases were not statistically significant despite the continuous upward trend. In MPS, relative conductivity sharply increased on 9 June, but no significant changes were observed during the third sampling on 7 July because the temperature increase was relatively small. By 6 August, relative conductivity significantly rose again with a larger temperature increase and excessive heat. Under high-temperature stress, compared with the initial control group, the relative conductivity of DRW increased the most (84.43%), and that of MPB increased the least (29.15%).

At Site 2, the relative conductivity of all varieties exhibited an overall upward trend with increasing stress temperatures (Figure 10b). At the onset of stress on 9 June, the relative conductivity of MPB, DR, MPR, and MPRR significantly increased compared to that of the initial control group. However, the increases in relative conductivity were not statistically significant for DRW, MPC, MPS, or EPY. On 7 July, the relative conductivity of MPB, DR, MPRR, and EPY showed no significant changes due to the relatively small temperature differences from the previous stage. In contrast, the relative conductivity of DRW, MPC, MPS, and MPR significantly increased. As the temperature further rose on 6 August, the relative conductivity of MPB, DRW, MPRR, and EPY considerably increased, whereas that of the other varieties showed smaller increases that were not statistically significant. At the end of the experiment, the relative conductivity of MPB increased the most (105.90%), while that of MPC increased the least (16.58%).

At Site 3, the relative conductivity of all varieties displayed an overall upward trend (Figure 10c). After the onset of stress, the relative conductivity of all varieties increased, except for that of MPRR, which decreased. Significant differences were observed in DR, MPC, MPS, and MPR. By the third sampling on 7 July, the relative conductivity of all varieties further rose as the temperature increased or excessive shading persisted. Significant differences were observed in MPB, DRW, DR, MPC, and MPRR. On 6 August, the relative conductivity of all varieties rose to the maximum values when the temperature further increased to 32 °C. Significant differences were observed in MPRR and EPY. At the end of the experiment, compared with the initial control group, the relative conductivity of MPC increased the most (85.40%), while that of EPY increased the least (15.05%).

A comprehensive comparison of the relative electrical conductivity of each cultivar across the three experimental sites revealed that most cultivars showed the highest relative electrical conductivity at Site 2 (Figure 10d). Furthermore, the results of a three-way ANOVA showed that, under natural high-temperature stress conditions, relative electrical conductivity was not significantly affected by cultivar, experimental site, or time (Figure S4). Overall, the effects of cultivar (F = 0.60), site (F = 2.11), time (F = 2.20), and their interactions were not significant. This suggests that, under the experimental conditions, the response of POD activity to high-temperature stress was relatively stable, and its changes did not significantly depend on the effects of genotype, environment, or stress duration.

### 3.6. Correlation Analysis of Physiological Indicators

**Figure 11:**
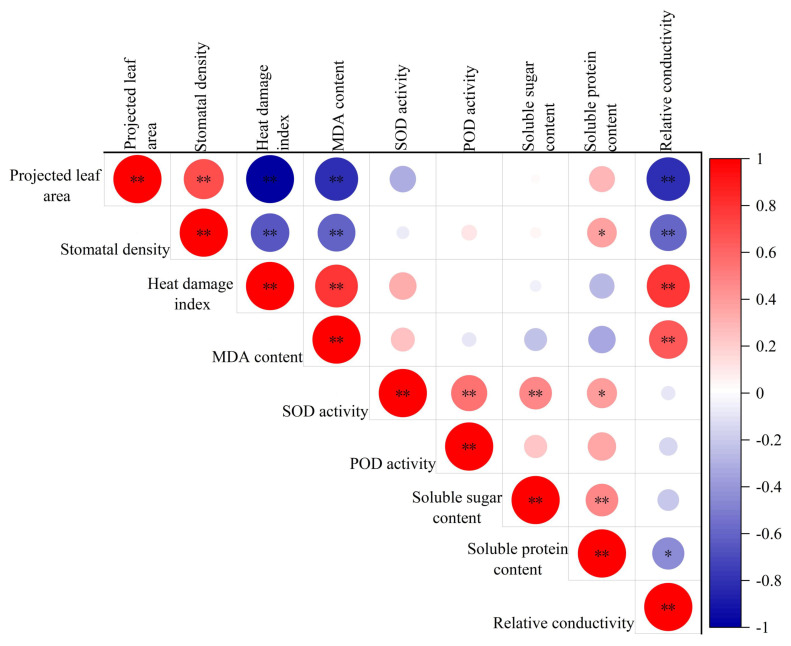
Correlation analysis of each index. Note: * significant correlation (*p* ≤ 0.05); ** very significant correlation (*p* ≤ 0.01).

A correlation analysis was performed on nine indicators for the eight *P. vulgaris* varieties tested (Figure 11). One or more significant or highly significant correlations were observed among the various indicators. The leaf area and stomatal density under heat stress exhibited a highly significant positive correlation (*p* < 0.01). The heat damage index, MDA content, and relative conductivity exhibited highly significant positive correlations with each other. Antioxidant enzyme activities (SOD and POD) presented a highly significant positive correlation with the soluble sugar and soluble protein contents. Additionally, SOD activity was highly significantly positively correlated with the soluble sugar content and significantly positively correlated with the soluble protein content. In contrast, both the leaf area and stomatal density showed highly significant negative correlations with the heat damage index, MDA content, and relative conductivity. The soluble protein content was significantly negatively correlated with relative conductivity. No significant correlations were observed among the other indicators. The correlation analysis results suggest that the heat tolerance of *P. vulgaris* is a complex trait that cannot be evaluated using a single indicator. Thus, a comprehensive evaluation combining multiple indicators should be conducted to accurately assess heat tolerance.

### 3.7. Heat-Responsive Gene Expression in P. vulgaris

**Figure 12:**
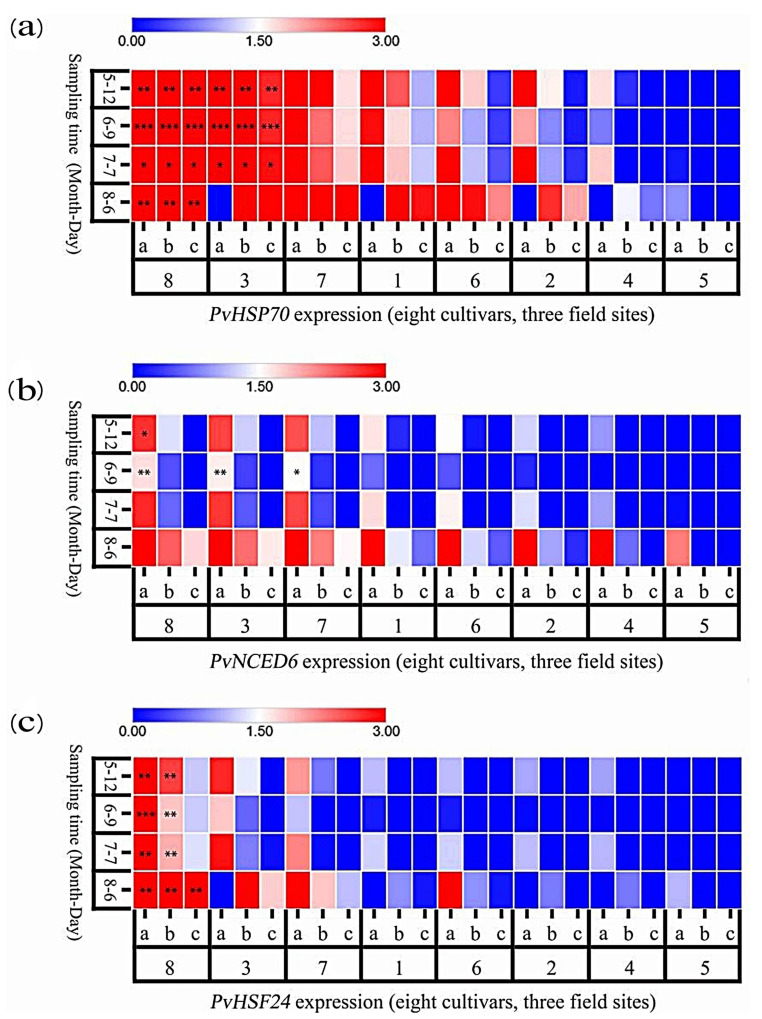
Heatmap of gene expression in eight cultivars of *P. vulgaris* at three field sites. (**a**) Site 1, (**b**) Site 2, and (**c**) Site 3. * Significant; ** highly significant; *** Extremely Highly Significant. Note: 1~8: Middle Punas Blue (MPB), Danova Rose White (DRW), Danova Red (DR), Middle Punas Crimson (MPC), Middle Punas Scarlet (MPS), Middle Punas Red (MPR), Middle Punas Rose Red (MPRR), and Early Punas Yellow (EPY).

As shown in Figure 12a, the expression of *PvHSP70* varied significantly among the cultivars and experimental sites. At Site 1, the expression of *PvHSP70* in MPB, DR, MPRR, and EPY was markedly upregulated in early June, with DR and EPY exhibiting highly significant differences from the control. By July, it declined in some cultivars, but it displayed an upward trend in DRW and MPR. On 6 August, it exhibited another significant increase in MPR, MPRR, and EPY, with EPY consistently maintaining high levels throughout the season. Overall, the expression levels of *PvHSP70* remained relatively high at Site 1 throughout the season, while those at Site 3 were generally lower, with only MPRR showing a significant increase at later stages and MPS showing the lowest levels.

The key ABA biosynthesis gene *PvNCED6* exhibited distinct expression patterns under heat stress (Figure 12b). At Site 1, the expression of *PvNCED6* in DR and EPY was notably significantly upregulated (*p* < 0.01) in the early stages, while that in MPRR showed significant differences (*p* < 0.05). It continued to increase in all cultivars in July, and it peaked in most of them in August. At Site 2, the expression levels across cultivars increased slowly with only minor changes. At Site 3, the overall expression was the lowest, with only EPY showing a late increase.

*PvHSF24* expression displayed an overall increasing trend across the three sites, reaching its peak in August (Figure 12c). It was highest at Site 1 and lowest at Site 3. Heat-tolerant cultivars (e.g., DR, MPRR, and EPY) generally showed higher expression than heat-sensitive cultivars (e.g., MPS and DRW). Notably, the expression level of *PvHSF24* was stronger than that of *PvHSP70*, suggesting that it plays a more critical role in heat stress responses.

### 3.8. Comprehensive Heat Tolerance Evaluation of P. vulgaris Varieties

Principal component analysis (PCA) was performed on the measured values of the nine indicators: leaf area, stomatal density, heat damage index, MDA content, SOD activity, POD activity, soluble sugar content, soluble protein content, and relative conductivity (Table S6). Dimensionality reduction and factor analysis identified three comprehensive components (*M*_1_, *M*_2_, and *M*_3_), whose cumulative contribution rate was 81.109%, with each component incorporating the relative coefficients (*X_kj_*) of the nine measured indicators previously mentioned (Table S7), as well as the comprehensive contribution rates (*P_k_*). These three components effectively represented most of the information from the original indicators.

Using the comprehensive evaluation method described in Section 2.4, the three principal components (*M*_1_, *M*_2_, and *M*_3_) were used as the basis for a comprehensive heat tolerance evaluation of the eight tested varieties.

Comprehensive indicator scores (*C*), membership function values (*U*), and comprehensive heat tolerance evaluation values (*D*) were calculated for each variety (Table 1). A larger *D* value indicates stronger heat tolerance. The results showed that, according to heat tolerance under natural high temperatures, the varieties ranked as follows: EPY > DR > MPRR > MPB > MPR > DRW > MPC > MPS (Table 1). Among them, the D values of EPY, DR, and MPRR were greater than 0.5, indicating relatively strong heat tolerance. EPY had the highest *D* value at 0.723, showing the best heat tolerance.

## 4. Discussion

The flowers and leaves of *P. vulgaris* are rich in vitamin C, minerals, and various natural antioxidant compounds; thus, they are commonly used as natural seasonings in salads, soups, and desserts. Their refreshing taste and vibrant color contribute to both their culinary appeal and nutritional value. In traditional Chinese medicine, *P. vulgaris* is regarded as an important herb for clearing heat and detoxifying the body by eliminating internal dampness and toxins [34]. *P. vulgaris* prefers cool temperatures, with an optimal growth range of 13–18 °C. It is highly sensitive to heat and strong light, thus requiring shading or placement in partially shaded environments during summer and autumn [35,36]. Consequently, summer survival has become a critical issue in the cultivation of *P. vulgaris*, limiting its application methods and geographical distribution [37]. Shading is the most cost-effective and easily implemented method for reducing temperatures in actual cultivation settings [38]. Considering the plant growth requirements, shaded forest environments in summer offer the most suitable and low-maintenance open-field conditions for the perennial cultivation of *P. vulgaris* [36]. Therefore, investigating the heat tolerance of different *P. vulgaris* varieties under natural high-temperature conditions, as well as identifying optimal forest-shading environments, is of great significance for expanding its range of applications and selecting superior cultivars.

Based on data from three experimental sites, significant differences were observed in the leaf area and stomatal density of *P. vulgaris* under high-temperature stress, which were closely related to ambient temperature. At Site 1, where the temperature was the highest and shading was minimal, leaf area declined most sharply, with that of most cultivars approaching zero, indicating severe inhibition of leaf growth. In contrast, at Site 3, where the shading rate was the highest and temperatures were the lowest, all cultivars maintained leaf areas above 20 mm^2^, and no complete stomatal closure or leaf desiccation-induced death occurred. This suggests that shading can alleviate the inhibitory effects of heat stress on leaf area by reducing leaf temperature. Under natural field conditions, the physiological responses of plants are often influenced by multiple environmental factors, including temperature, light intensity, and air humidity. However, in this study, ultraviolet radiation levels were consistent between the three sites (Table S1), and the air humidity was similar (Figure S2). The most significant environmental gradient observed was the temperature variation. Therefore, the leaf growth changes observed in this study were primarily associated with the increasing temperature between different sites. Previous studies have shown that high-temperature stress slows leaf growth, mainly due to reduced cell elongation and decreased transpiration efficiency [39]. Regarding stomatal density, under high temperatures, plants often close stomata to reduce water loss and maintain water balance, which, in turn, limits CO_2_ uptake and suppresses photosynthesis [40]. Stomatal density generally declined in the early stages of heat stress. However, some heat-tolerant cultivars, such as EPY and DR, were able to maintain a relatively high stomatal density in the later stages. In contrast, cultivars such as DRW, MPC, and MPS exhibited complete stomatal closure across multiple sites, indicating high sensitivity to heat stress. Overall, the coordinated changes in leaf area and stomatal density reflect the physiological regulation patterns and germplasm differences in *P. vulgaris* under natural high-temperature conditions. Moderate shading effectively reduces leaf temperature, delays stomatal closure, and helps maintain leaf function [41]. In fact, for heat-sensitive species such as *P. vulgaris*, even deep shading can provide substantial protection under extreme summer conditions. Under natural field conditions, plant growth is often influenced by a combination of multiple environmental factors. At Site 3, the extremely high shading rate, combined with frequent rainfall, created a relatively humid microclimate environment. Although this significantly reduced heat stress, it also increased the likelihood of disease occurrence. However, this phenomenon is also one of the complex environmental effects found in natural ecosystems [42].

Regarding the temperature variations observed at the experimental sites, the cooling effect was significantly enhanced with increasing shading rates, and temperatures during the four samplings exhibited significant differences among the sites. The impact of high-temperature stress on plants was most directly reflected in their external morphology, as the heat damage index was negatively correlated with plant heat tolerance [43]. Observations of heat damage severity at the experimental sites revealed that most plants were not damaged or exhibited only mild heat damage, such as slight yellowing at the leaf edges, when the average temperature remained below or around 30 °C, allowing for normal growth. However, the severity of heat damage significantly increased as temperatures consistently exceeded 30 °C and approached 35 °C, with the plants displaying poor growth and larger areas of leaf scorching. Many plants experienced severe dehydration, wilting, or even death when temperatures rose to nearly 40 °C, suggesting that, to maintain normal growth in the cultivation and application of *P. vulgaris,* summer environmental temperatures should not exceed 30 °C. At Site 3, the shading rate reached 98%, and this excessive shading led to elevated air humidity. When combined with frequent rainfall during the wet season, this created a higher risk of disease occurrence [42]. Nevertheless, the heavily shaded environment effectively reduced both the light intensity and leaf temperature, thereby offering important protection for *P. vulgaris* during periods of intense summer heat. Therefore, in practical cultivation, factors such as summer survival, disease susceptibility, and local climatic conditions should be carefully considered to optimize the growth environment [44,45].

Under high-temperature stress, plant cell and organelle membranes were damaged [46], leading to lipid peroxidation and the accumulation of MDA [47]. Simultaneously, the increased cell membrane permeability resulted in electrolyte leakage and, thus, an increase in relative conductivity. Therefore, the MDA content and relative conductivity were negatively correlated with plant heat tolerance. In this study, the MDA content and relative conductivity of all varieties at Sites 1 and 2 exhibited an upward trend with increasing temperatures and prolonged high-temperature stress. This is consistent with previous findings on the heat tolerance of *P. forrestii* [48] and *Vitis vinifera* L. [49]. The temperatures at Site 1 were significantly higher than those at Site 2 in all sampling periods. Consequently, the average increases in the MDA content and relative conductivity at Site 1 were higher than those at Site 2, further confirming that higher temperatures resulted in a greater MDA content, higher relative conductivity, and more severe plant damage [50]. At Site 3, the cooling effect was significant, with temperatures being below 30 °C during the first three samplings and reaching a maximum of only 32 °C on 6 August. Although the temperatures did not significantly impact the plants, excessive shading inhibited their growth and development [51]. Under low-light stress, the degree of membrane lipid peroxidation increased [52], and the MDA content and relative conductivity of all varieties showed an overall upward trend, in line with the findings of Yu et al. [53] on *Liquidambar formosana* seedlings.

Under high-temperature stress, reactive oxygen species (ROS) excessively accumulate. As key protective enzymes in plants, the activities of SOD and POD can increase within a certain temperature range to eliminate excess ROS, and this process is positively correlated with heat tolerance [54]. In this study, the SOD activity of the plants at Sites 1, 2, and 3 exhibited an overall upward trend, reaching the peak in most varieties during the fourth sampling, where it was significantly higher than that of the control group. In other words, the SOD enzyme activity of the plants increased to enhance heat tolerance under high-temperature stress. These findings are consistent with those of previous studies on rice seedlings [55]. The increases in the SOD activity of all varieties at each site were averaged, and Site 2 showed the largest average increase, followed by Sites 1 and 3. This suggests that SOD activity increased to counteract stress within a certain high-temperature range [39]. When temperatures exceed a plant’s defensive limits, however, enzyme structures may be altered, or enzyme expression may be suppressed, leading to a decrease in SOD activity [56]. Therefore, it was determined that the moderately high temperature and shading at Site 2 contributed the most to the increase in SOD activity. Although the temperatures at Site 3 were relatively low, excessive shading resulted in low-light inhibition, affecting enzyme synthesis. The POD activity of the varieties across sites did not show a uniform trend due to differences in plant response speed and temperature tolerance ranges. The results suggest that elevated POD activity commonly indicates oxidative stress or the initiation of defense mechanisms [57]. Although the POD activity of most varieties increased to resist high temperatures, a few varieties, such as EPY, demonstrated strong environmental adaptability, and their POD activity decreased after adapting to heat stress [58]. Consequently, at the end of the experiment, the POD activity of some varieties was higher than that of the initial control group and lower than that of other groups. In summary, SOD activity played a more significant defensive role than POD activity under high-temperature stress, acting as the primary defense mechanism. These results align with the findings of Qiu et al. [59] regarding the heat tolerance of *Rubus idaeus* (red raspberry).

Under high-temperature stress, plants undergo severe cellular water loss and an osmotic imbalance between the interior and exterior of cells [60]. Soluble sugar and soluble protein concentrations increase to raise cell sap concentrations and maintain osmotic balance [61]. In this study, the soluble sugar content at Sites 1 and 2 exhibited an overall upward trend. At Site 2, the soluble sugar contents of all varieties reached their maximum values during the fourth sampling, exceeding those of the control group. At Site 1, most varieties showed an increase in soluble sugar content compared to the initial control group by the end of the experiment. This suggests that the increase in soluble sugar content under high-temperature stress enhanced plant heat tolerance, consistent with previous studies on *Rhododendron* and *P. forrestii* [54]. Although the temperature at Site 3 was lower, excessive shading affected photosynthesis, thereby inhibiting the synthesis of soluble sugars, which are an essential product of this process. Consequently, the soluble sugar content showed a minimal increase, and, in many varieties, it was lower than in the control group at the end of the experiment. These results are in line with the findings of Yu et al. [53]. The soluble protein content of all varieties across the experimental sites followed a decreasing-then-increasing trend. Soluble protein synthesis was inhibited during the initial stress period. Soluble protein content increased to counteract stress-induced damage as plants adapted to the environment [62]. Overall, Site 2 exhibited the highest average increases in both soluble sugar and soluble protein contents. Hence, moderate shading under high-temperature stress is beneficial for maintaining normal growth and mitigating stress damage in *P. vulgaris*, whereas excessive or insufficient shading is detrimental to plant survival.

In recent years, molecular biology has made significant progress in elucidating the genetic and molecular mechanisms underlying plant heat tolerance. Studies have shown that heat shock proteins, transcription factors, and signal transduction-related genes play central roles in responses to high-temperature stress. For example, in peony, the *PSHSP70* gene is significantly induced under high-temperature conditions [63], and transcriptomic analyses have identified multiple heat shock protein family members associated with heat stress responses [64]. In the genus *P. vulgaris*, small heat shock protein genes such as *PHSP21.4* and *PHSP17*.1 have been found to play important roles in heat stress responses and are closely associated with heat tolerance, drought resistance, and salt tolerance [65]. In addition, previous studies have demonstrated that plant heat tolerance is also jointly regulated by molecular and hormonal pathways: heat shock proteins (e.g., *HSP70* and *HSP90*) protect proteins and membrane structures under high-temperature stress; antioxidant enzymes (e.g., SOD and CAT) play key roles in mitigating oxidative damage caused by the accumulation of reactive oxygen species; and phytohormones (e.g., ABA, ethylene, and cytokinins) contribute to stomatal regulation and stress adaptation [66,67,68]. The reductions in leaf area, alterations in stomatal characteristics, and differences in oxidative damage observed in this study are likely to be closely related to these molecular regulatory mechanisms. These findings are also consistent with the hormone-mediated regulatory pathways summarized by Li et al. [69]. In fact, plant hormones such as ABA, SA, JA, CK, ET, and BR play central roles in heat tolerance by regulating ROS homeostasis, antioxidant metabolism, and HSP expression, and they engage in complex signaling interactions [70]. Integrating our field experiment results with these established mechanisms helps strengthen the physiological interpretation of the heat tolerance of *P. vulgaris*.

Under field high-temperature stress, different genes exhibited both distinct and complementary expression patterns. *PvHSP70* expression was significantly upregulated in heat-tolerant cultivars such as EPY and MPRR, especially at Site 1. This finding supports the classical role of heat shock proteins as molecular chaperones in protecting protein stability under heat stress [71,72,73]. In contrast, *PvNCED6*, the key gene for ABA biosynthesis, showed a gradual but sustained increase across sites, suggesting that *PvNCED6* may participate in early ABA-mediated responses to heat stress and may contribute to plant heat adaptation through stomatal regulation and stress signaling pathways [74]. Importantly, the transcription factor *PvHSF24* exhibited the highest expression, approximately twice that of *PvHSP70*, and maintained high levels in later stages. This may indicate that HSFs are more likely to function as upstream regulators during heat stress, participating in the regulation of downstream stress-responsive genes [75]. Moreover, differences in expression patterns between heat-tolerant and -sensitive cultivars (e.g., EPY vs. MPS) further support the notion that the intensity of molecular activation is closely related to phenotypic thermotolerance [76]. Overall, these results provide preliminary evidence that HSF-, HSP-, and ABA-related pathways may participate in the heat stress response of *P. vulgaris* [77]. Among them, *PvHSF24* represents a candidate gene worthy of further investigation and may have potential applications in future breeding or molecular improvement.

Consistent with physiological trait evaluations, the expression patterns of heat-responsive genes further confirmed differences in thermotolerance among cultivars. In particular, *PvHSP70* and *PvHSF24* were strongly upregulated in heat-tolerant cultivars such as EPY and MPRR, consistent with their performance under heat stress, including a larger leaf area, higher stomatal density, and lower heat index [78]. Conversely, in sensitive cultivars such as MPS, MPC, and DRW, the induction of *PvHSP70* and *PvHSF24* was weaker or delayed, corresponding to severe leaf damage and aggravated heat injury, suggesting that insufficient molecular responses are a key reason for their poor thermotolerance [79]. Additionally, the gradual increase in *PvNCED6* expression across cultivars and sites suggests that ABA-mediated stomatal regulation and osmotic adjustment play critical roles in the long-term adaptation of heat-tolerant cultivars [80]. Overall, the differential gene expression patterns were highly consistent with physiological assessments, further supporting the reliability of the integrated thermotolerance ranking.

In this study, the correlation among nine measured indicators was analyzed, revealing that the heat damage index, MDA content, and relative conductivity were negatively correlated with plant heat tolerance. These three indicators also exhibited highly significant positive correlations with each other. In contrast, plant heat tolerance, leaf area, and stomatal density were highly positively correlated with each other. Additionally, soluble sugars and soluble proteins (osmotic regulatory substances), as well as the activities of SOD and POD (protective enzymes), were highly significantly positively correlated [81], further validating the reliability of the correlation analysis. The PCA-based ranking of heat tolerance was consistent with the observed phenotypic traits of the experimental plants, confirming that the heat tolerance of *P. vulgaris* is a composite trait influenced by multiple indicators [82]. Thus, comprehensive evaluations should be performed based on multiple indicators. The heat tolerance of the eight *P. vulgaris* varieties was comprehensively evaluated by combining principal component analysis with the membership function method. According to heat tolerance, the varieties were ranked from highest to lowest as follows: EPY > DR > MPRR > MPB > MPR > DRW > MPC > MPS. This ranking aligns with the observed heat damage. This indicates that the heat damage index is the most direct indicator of heat damage severity and plays a significant role in assessing heat tolerance [83]. Therefore, it should carry the highest weight when examining multiple indicators. Varieties such as EPY, DR, and MPRR (*D* > 0.5) demonstrated strong heat tolerance, with favorable phenotypic performance under high temperatures. These varieties should be prioritized for breeding and improvement for extensive application in high-temperature regions during summer. Varieties such as MPR and MPB demonstrated moderate heat tolerance and can be used as moderately shaded groundcover plants under forest canopies to enhance landscape diversity. Varieties with poor heat tolerance, such as DRW, MPC, and MPS, are not recommended for application in high-temperature regions during summer. To further assess the stability of variety performance under different environmental conditions, PCA was performed on the data obtained from the three sites. The results indicated that heat-tolerant varieties showed relatively consistent rankings across different environmental conditions. For example, EPY, DR, and MPRR consistently ranked among the top four for heat tolerance at all three sites, suggesting that these varieties exhibit stable heat tolerance across different microclimate conditions. In contrast, varieties with relatively weaker heat tolerance showed some variation in their rankings across the different sites. For instance, at Site 1, which experienced more severe heat stress, MPC and MPS were ranked seventh and eighth, respectively; however, at Site 3, which had better shading conditions, they climbed to second and fifth place. This indicates that varieties with weaker heat tolerance are positively influenced by environmental factors under stress conditions. Therefore, the stability of the heat-tolerant varieties across different sites provides partial support for the reliability of the heat tolerance evaluation results in this study, while the ranking differences for the heat-sensitive varieties reflect the potential effects of cultivar × site interactions on plant performance in complex natural environments.

According to the three-way ANOVA results, different types of indicators in *P. vulgaris* exhibited significantly different regulatory patterns under natural high-temperature stress. Morphological and comprehensive damage indicators such as leaf area, stomatal density, and heat damage index all revealed significant “site × time” interaction effects, indicating that their changes were essentially influenced by the combined effects of environmental factors and time accumulation. Especially during the sustained high-temperature period in August, the site effect reached its peak, suggesting that environmental conditions play a core role in determining the degree of plant damage [84]. Consistent with this, all cultivars performed worst at Site 1, which had the lowest shading and highest temperature, while maintaining better growth in the forest canopy environment (Site 3). This further verifies that environmental conditions are a crucial factor affecting the summer survival of *P. vulgaris* [85]. In contrast, some physiological regulatory indicators demonstrated more complex response characteristics to high-temperature stress. SOD activity and stomatal-related traits also suggested significant “site × time” interaction effects, reflecting the distinct stages and environmental dependence of their regulatory processes. This indicates the dynamic adaptive capacity of plants to environmental changes during the stress process. In contrast, MDA, POD, and relative electrical conductivity failed to exert significant interaction effects, and relative electrical conductivity showed the weakest discriminatory ability among the factors under the experimental conditions. In other words, it reflects the overall stress level rather than fine-tuned regulatory processes under natural high-temperature conditions [86,87]. Notably, soluble protein content was primarily influenced by the cultivar effect, indicating that this indicator reflects the intrinsic characteristics of the genotype rather than a highly sensitive response to environmental changes. Overall, the “site × time” interaction was the main source of variation explaining changes in several crucial indicators, while the cultivar effect and its interaction with the experimental site contributed relatively less. In short, under continuous high-temperature stress conditions, environmental conditions and the cumulative effect of time play a dominant role in the physiological response of *P. vulgaris* [88].

In this study, Site 1 resulted in the most severe heat damage due to low shading and higher ambient temperatures, which significantly affected plant growth and development. In contrast, Site 2 had a moderate level of shading, resulting in limited cooling effects under partial shade conditions and a moderate alleviation of heat stress. Site 3, with the highest shading rate, demonstrated a remarkable cooling effect. A comprehensive analysis of various physiological indicators at the three experimental sites revealed that Site 3 differed significantly from the other sites, with a clear decreasing trend in parameters. This demonstrates that Site 3 significantly improved the heat response of *P. vulgaris* cultivars, making it the optimal environmental condition for their summer cultivation. In practical landscape applications, cultivating *P. vulgaris* as an ornamental groundcover plant in shaded woodland areas—such as dense forest understory grasslands—not only supports summer survival but also significantly reduces cooling and maintenance costs, thereby offering an ideal solution for perennial open-field cultivation [89]. In contrast, small trees with low shading provide poor cooling effects and fail to effectively reduce heat stress on *P. vulgaris* [90]. Meanwhile, the limited ameliorative effect of sparse forests results in unsatisfactory summer survival performance of *P. vulgaris*. Neither of these two environments is optimal for the open-field cultivation of this species.

Nevertheless, this study has certain limitations. It was restricted to a single-season observation of eight cultivated varieties and did not include wild germplasm or multi-year trials. Moreover, trait observations mainly focused on leaves and physiological parameters, without a systematic evaluation of floral or root traits. Future studies will extend to a broader range of germplasm resources and multi-season validations, and they will integrate molecular and hormonal analyses to provide a more comprehensive understanding of the heat tolerance mechanisms of *P. vulgaris*.

## 5. Conclusions

This study assessed the heat tolerance of eight widely cultivated *P. vulgaris* varieties under three shaded forest environments with varying shading rates. The evaluation included the heat damage index and measurements of eight physiological indicators: plant heat tolerance, leaf area, MDA content, SOD activity, POD activity, soluble sugar content, soluble protein content, and relative conductivity. A correlation analysis was performed on these indicators, and a comprehensive heat tolerance evaluation was conducted using the membership function method.

The study revealed a highly significant positive correlation between leaf area and stomatal density under heat stress (*p* < 0.01). A highly significant positive correlation was also observed among the heat damage index, MDA content, and relative conductivity. In response to heat stress, SOD activity and soluble sugar and soluble protein contents increased to mitigate damage, while POD activity showed no consistent trend. There were highly significant positive correlations among the activities of protective enzymes, as well as among the levels of osmotic adjustment substances. Additionally, both leaf area and stomatal density showed highly significant negative correlations with the heat damage index, MDA content, and relative conductivity. As a result of this evaluation, the tested varieties were ranked according to heat tolerance as follows: EPY > DR > MPRR > MPB > MPR > DRW > MPC > MPS. Among these, the heat tolerance of EPY, DR, and MPRR was the strongest, with that of EPY being the best. This study provides significant academic value and practical implications for evaluating the heat tolerance and understanding the heat-induced physiological responses of *P. vulgaris*. It also offers an important reference for advancing the selection of heat-tolerant varieties.

The expression of *PvHSP70* and *PvHSF24* was significantly upregulated in heat-tolerant cultivars, while that of *PvNCED6* showed a sustained increase with rising temperatures. The expression trends of these genes were consistent with physiological trait analyses, further validating the reliability of the observed thermotolerance differences among the cultivars.

A three-way ANOVA revealed that the physiological response of *P. vulgaris* under natural high-temperature stress exhibited a pronounced environment-dominated characteristic. Site, time, and “site × time” interaction effects are crucial factors determining the degree of plant damage and summer survival performance. In contrast, the cultivar and “cultivar × site” interaction effects were relatively weak. This result suggests that, under natural open-field high-temperature conditions, improving environmental conditions, especially increasing shading and stabilizing temperature, is more effective in enhancing the summer survival of *P. vulgaris* than simply relying on cultivar differences. This study provides clear experimental evidence supporting the selection of suitable open-field cultivation environments for *P. vulgaris* during the summer.

Additionally, all *P. vulgaris* cultivars showed the best summer survival performance at Site 3 under optimal shading conditions. Therefore, dense forest understory grasslands are the most suitable environment for the open-field summer cultivation of *P. vulgaris*. Overall, the findings of this study not only provide a theoretical basis for the screening and utilization of heat-tolerant cultivars but also offer molecular insights to support future breeding programs aiming to improve the thermotolerance of *P. vulgaris*.

## Figures and Tables

**Table 1:** Comprehensive evaluation of heat resistance of various varieties.

Breed Number	*C*_1_	*C*_2_	*C*_3_	*U*_1_	*U*_2_	*U*_3_	*D*	Heat Resistance Order
1	13.471	5.586	2.021	0.397	0.464	0.661	0.447	4
2	20.069	4.491	1.953	0.000	0.750	0.647	0.304	6
3	12.513	3.533	1.322	0.454	1.000	0.510	0.629	2
4	17.591	7.365	3.112	0.149	0.000	0.898	0.186	7
5	18.177	7.197	1.760	0.114	0.044	0.605	0.147	8
6	12.752	7.227	3.583	0.440	0.036	1.000	0.377	5
7	8.639	6.171	2.209	0.687	0.311	0.702	0.573	3
8	3.439	5.588	−1.029	1.000	0.464	0.000	0.723	1

Note: 1~8: Middle Punas Blue (MPB), Danova Rose White (DRW), Danova Red (DR), Middle Punas Crimson (MPC), Middle Punas Scarlet (MPS), Middle Punas Red (MPR), Middle Punas Rose Red (MPRR), and Early Punas Yellow (EPY).

## Data Availability

The original contributions presented in this study are included in the article/Supplementary Material. Further inquiries can be directed to the corresponding author.

## Supplementary Materials

The following supporting information can be downloaded at https://www.mdpi.com/article/10.3390/plants15071000/s1, Figure S1: Variation trend of degree day of ground temperature in each experiment; Figure S2: Variation trend of degree day of Relative Humidity in each experiment; Figure S3: Rainfall Statistics for Experimental Sites; Figure S4: F-values Heatmap (Three ANOVA); Figure S5: Representative stomatal micrographs of *P. vulgaris* under natural high temperature; Figure S6: The morphological variation trend of *P. vulgaris* under natural high temperature. Table S1: Environmental parameters across experimental sites (mean ± SD); Table S2: Sampling time and temperature of each test site (°C); Table S3: The shading rate at each experimental site; Table S4: Transcript IDs and primer sequences used for qRT-PCR; Table S5: Changes in stomatal density of *P. vulgaris* varieties at each experimental site; Table S6: Characteristic value, contribution rate and weight value; Table S7: Component Matrixa.
