# Supplementary figures and images for: Physiological Responses and Heat Tolerance Evaluation of Eight Varieties of Primula vulgaris Under Natural High Temperatures
Source: Plants (Basel). 2026 Mar 25;15(7):1000. doi: 10.3390/plants15071000 (PMC13074257; doi:10.3390/plants15071000)

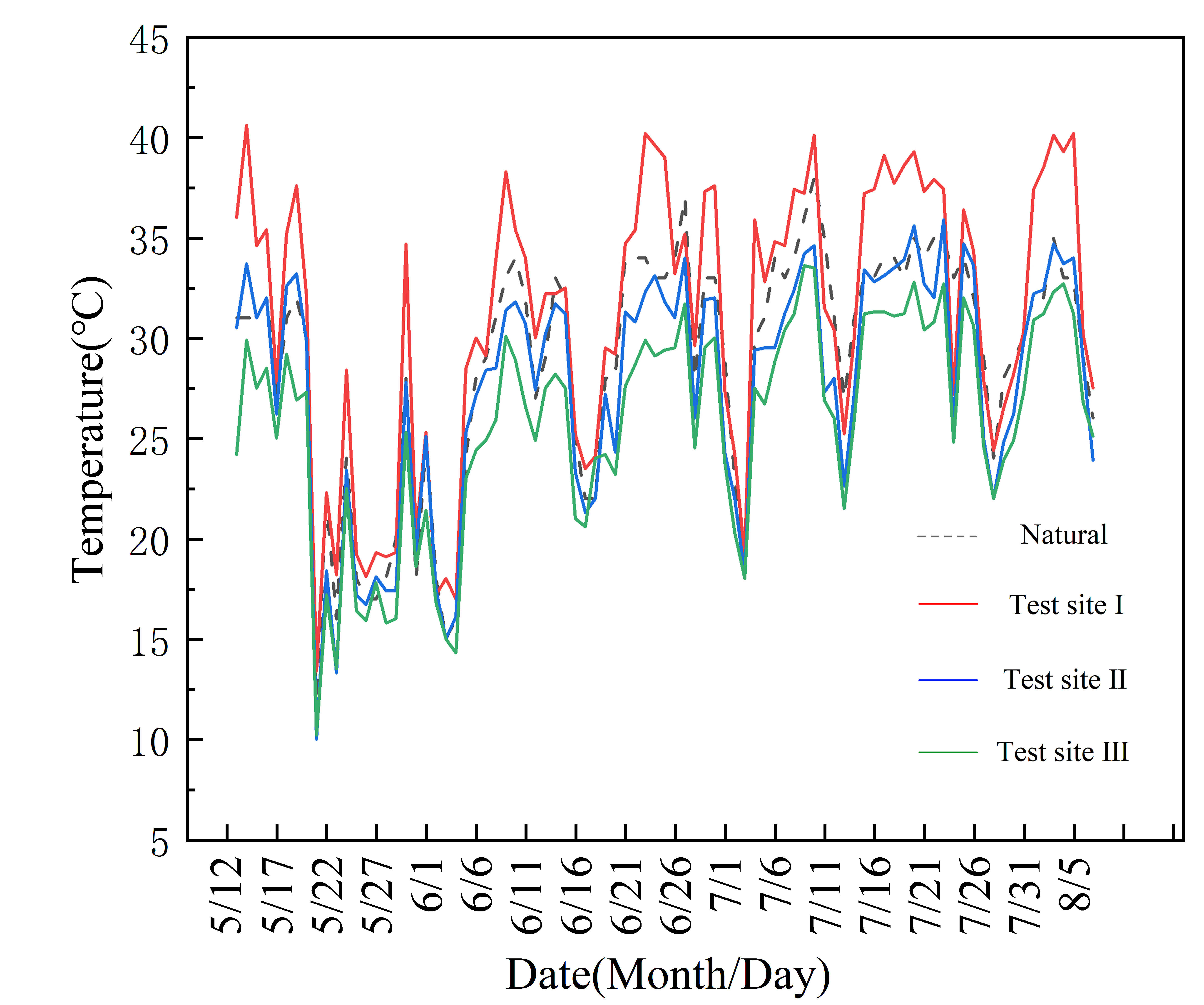

Supplement: Supplementary file 1 [file plants-15-01000-s001.zip › 01Fig.S1 Variation trend of degree day of ground temperature in each experiment.jpg]

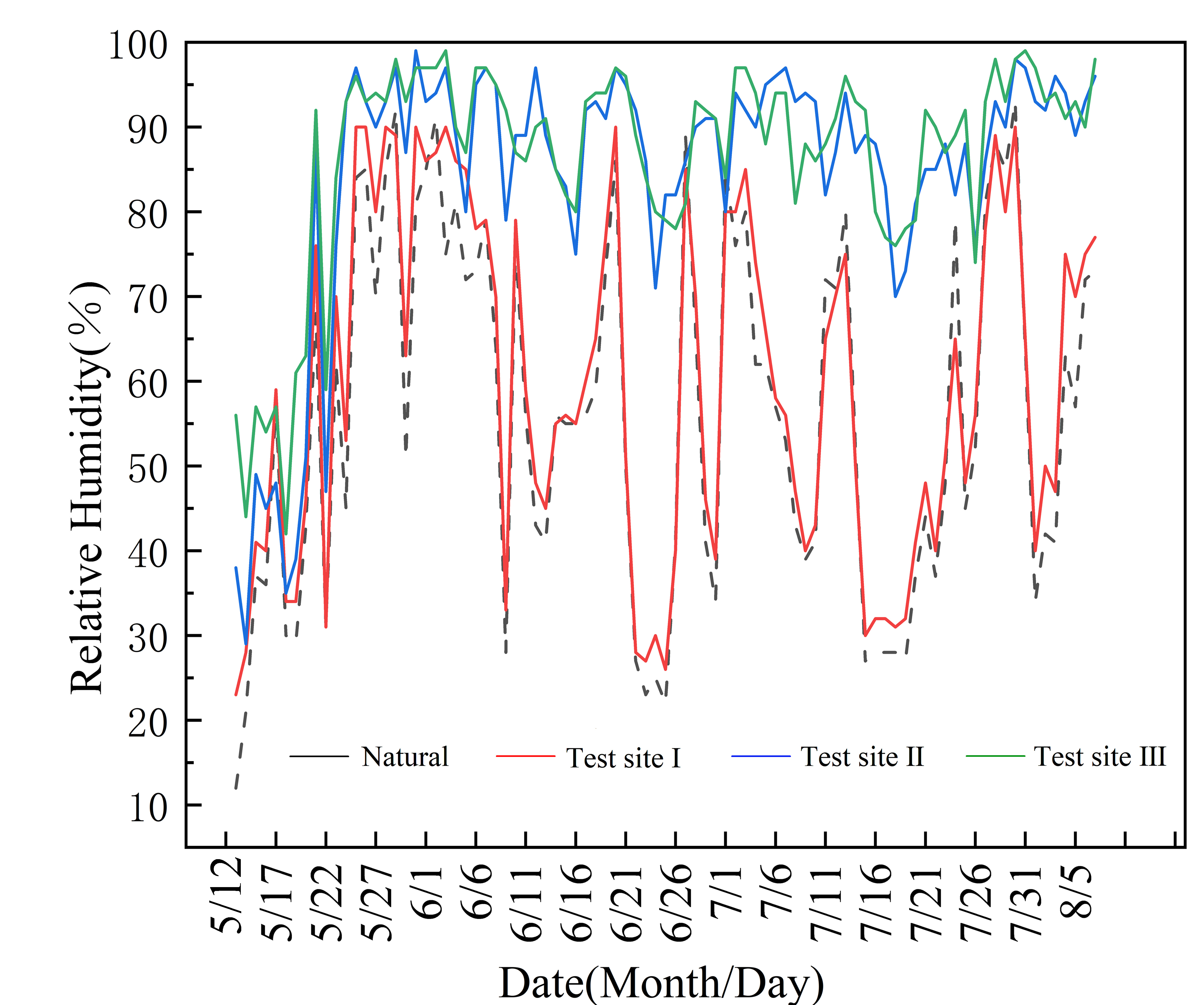

Supplement: Supplementary file 1 [file plants-15-01000-s001.zip › 02Fig. S2 Variation trend of degree day of Relative Humidity in each experiment.jpg]

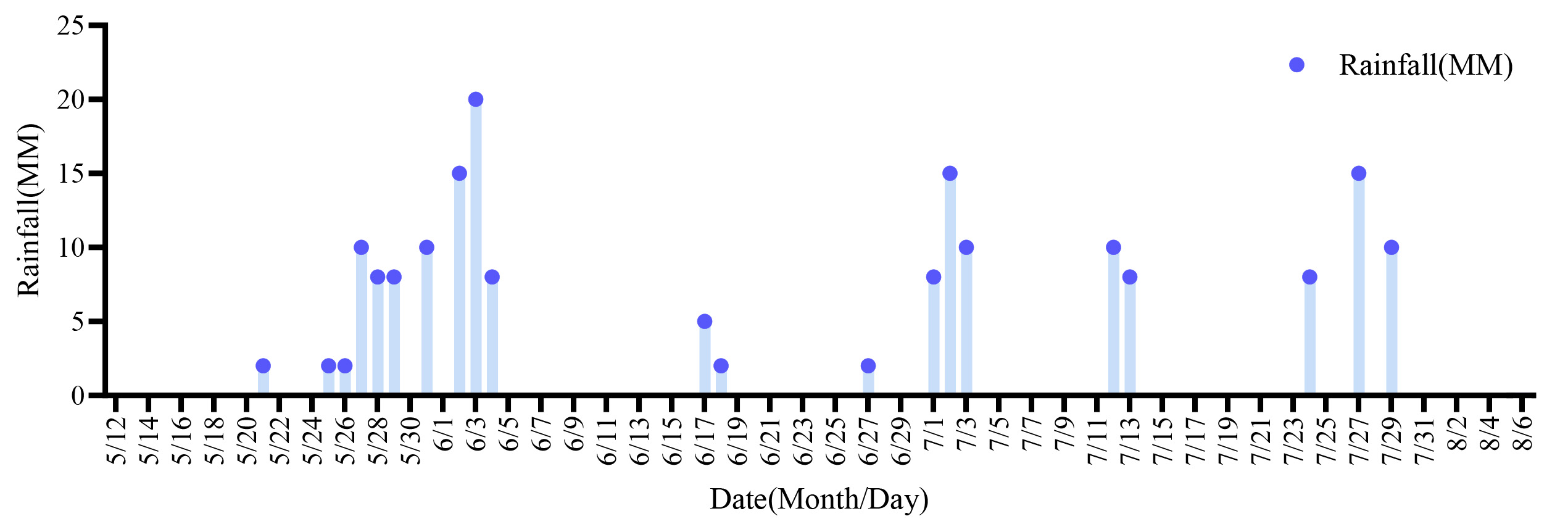

Supplement: Supplementary file 1 [file plants-15-01000-s001.zip › 03Fig. S3 Rainfall Statistics for Experimental Sites.jpg]

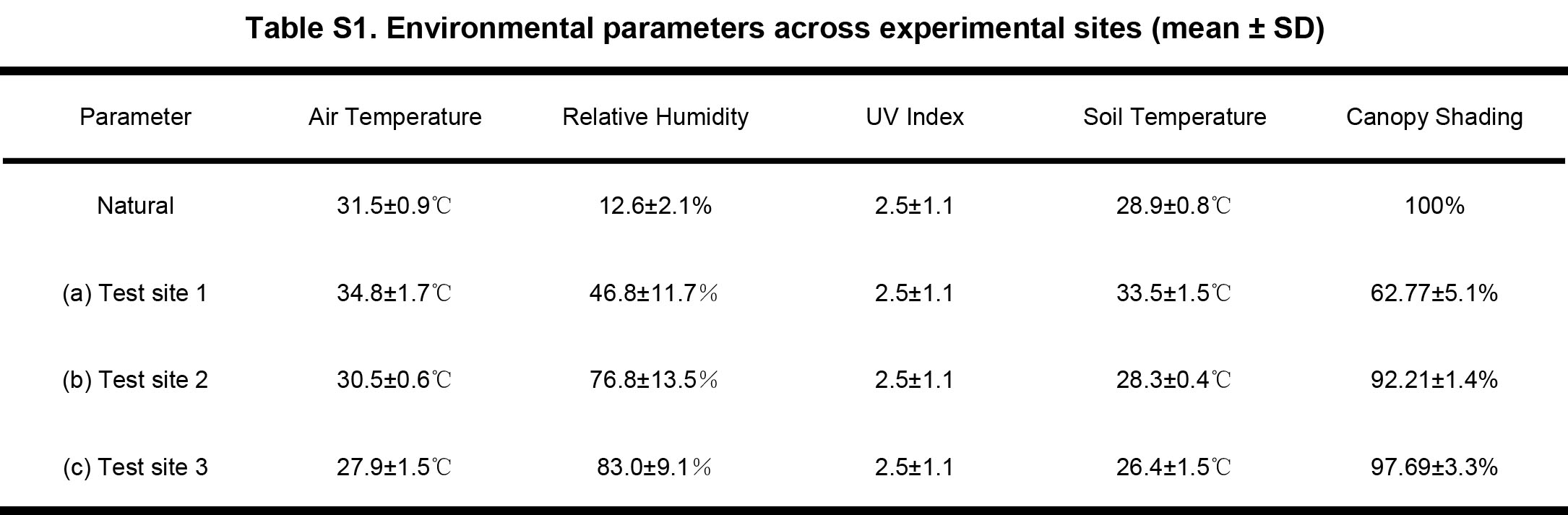

Supplement: Supplementary file 1 [file plants-15-01000-s001.zip › 07Tab. S1 Environmental parameters across experimental sites (mean ± SD).jpg]

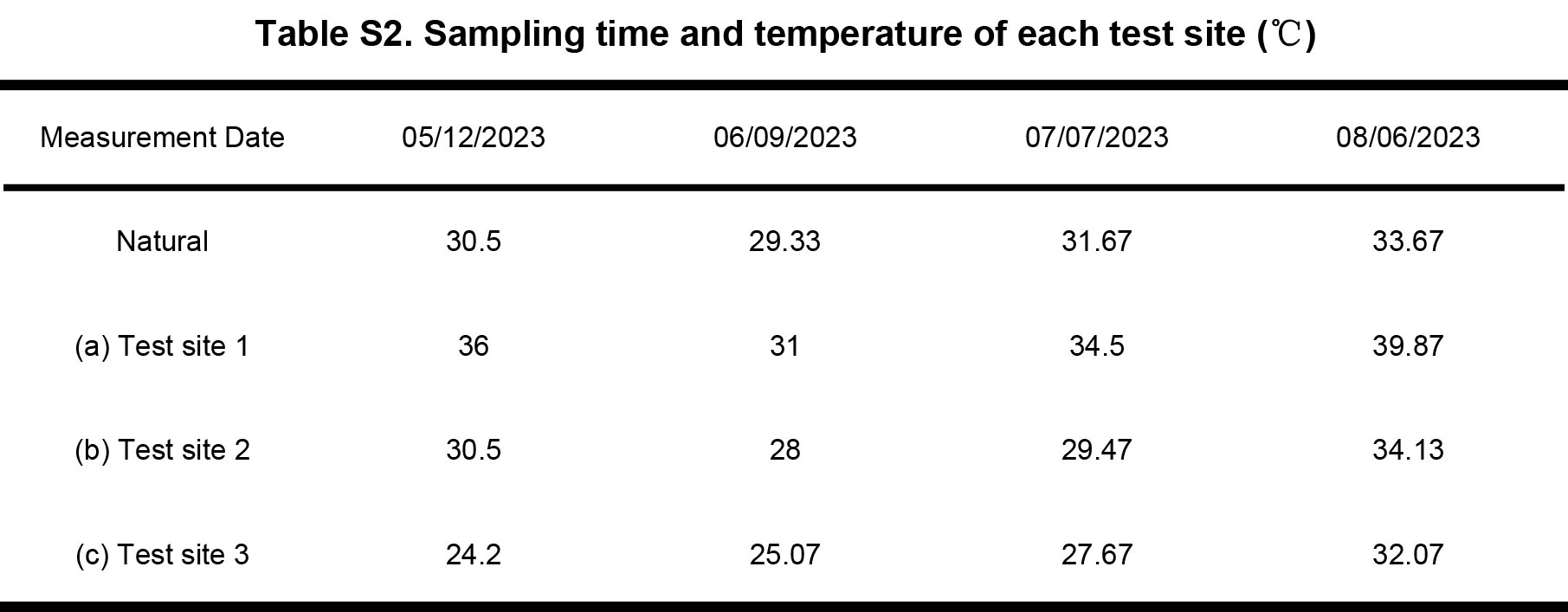

Supplement: Supplementary file 1 [file plants-15-01000-s001.zip › 08Tab. S2 Sampling time and temperature of each test site (°C).jpg]

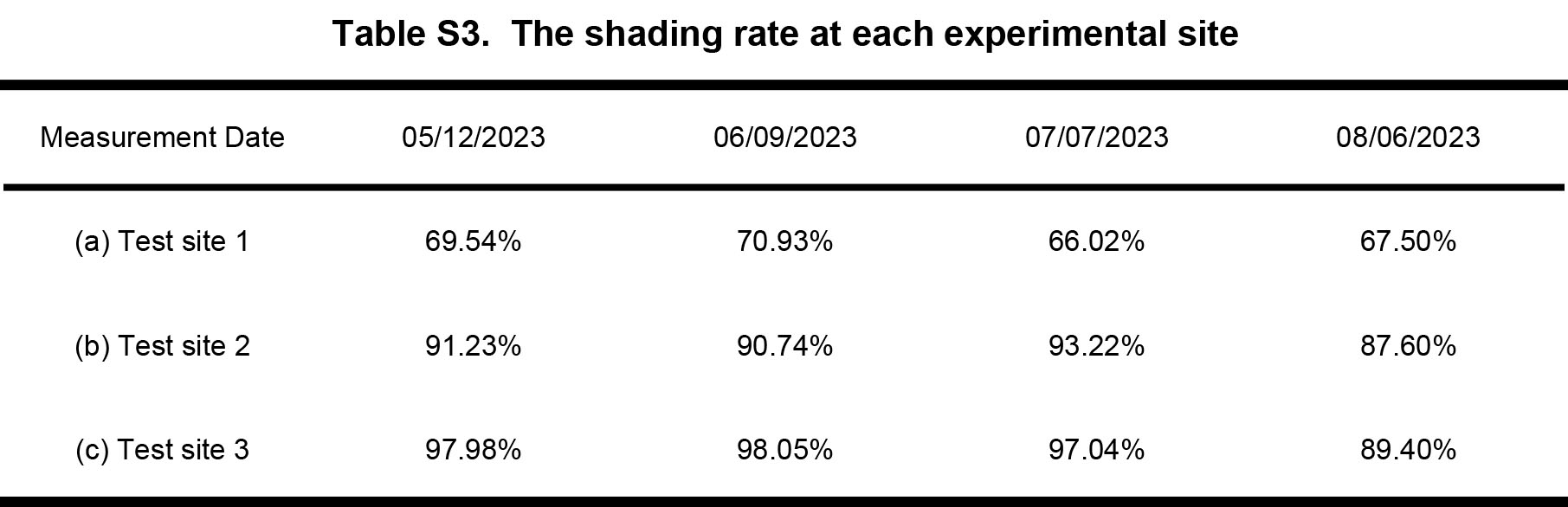

Supplement: Supplementary file 1 [file plants-15-01000-s001.zip › 09Tab. S3 The shading rate at each experimental site.jpg]

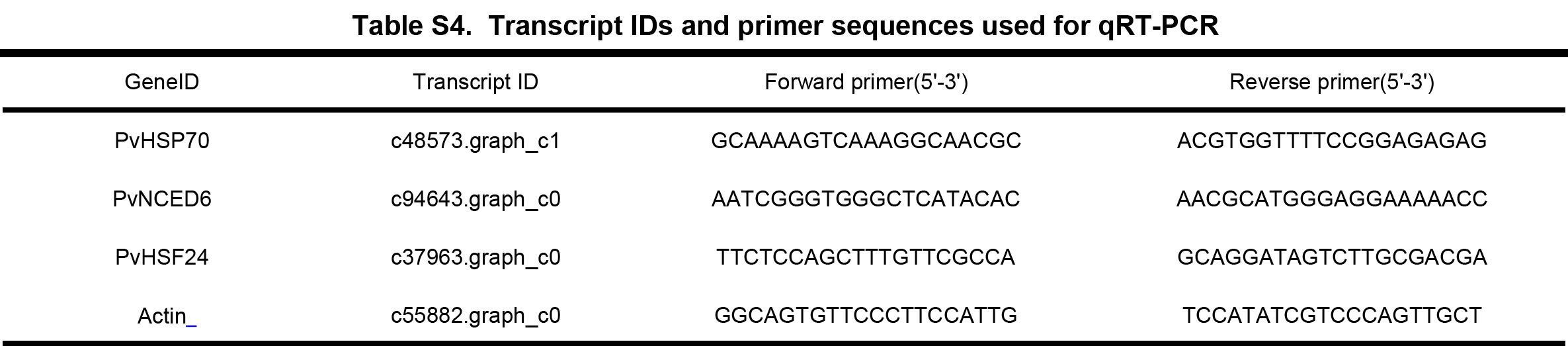

Supplement: Supplementary file 1 [file plants-15-01000-s001.zip › 10Tab. S4 Transcript IDs and primer sequences used for qRT-PCR.jpg]

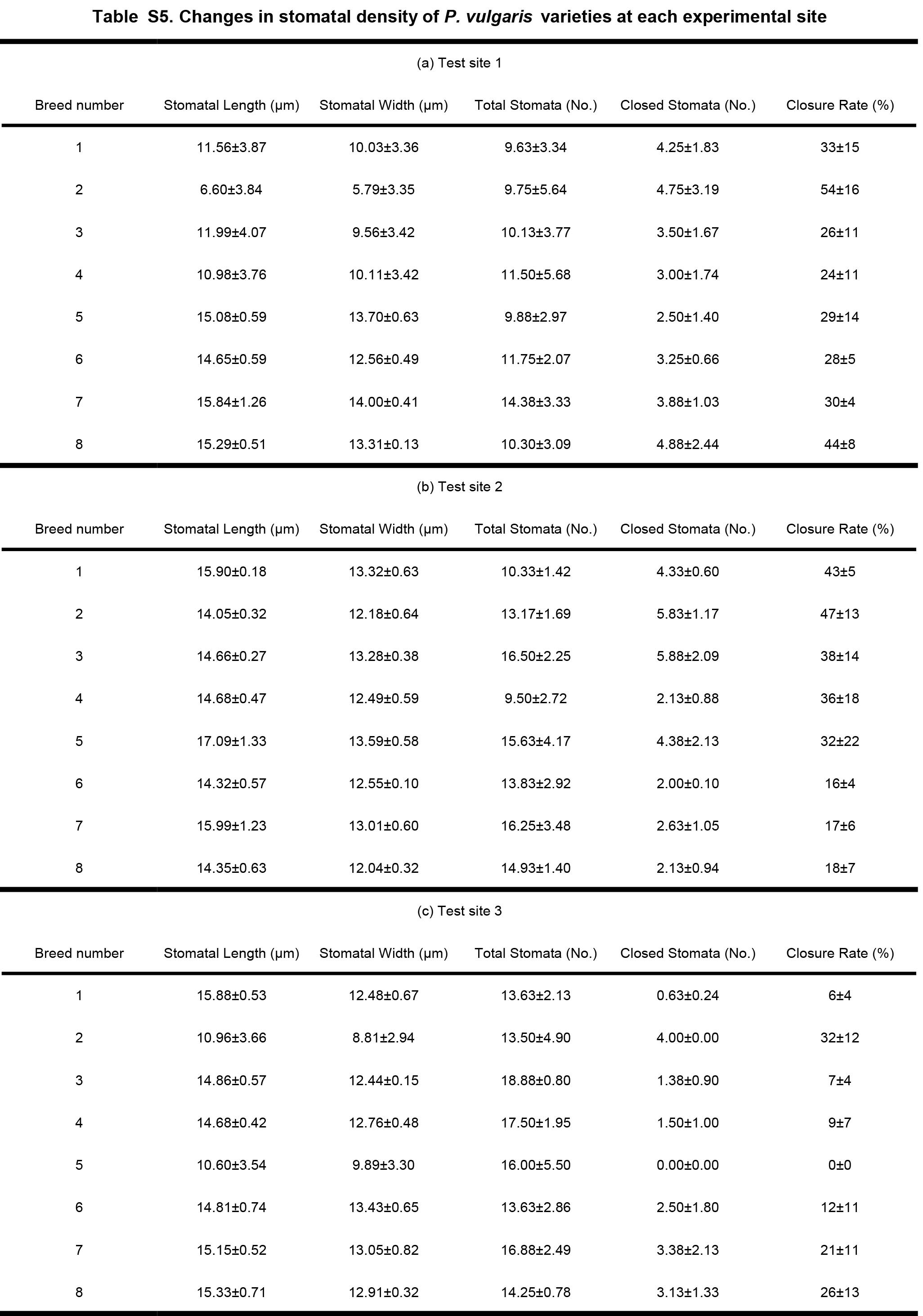

Supplement: Supplementary file 1 [file plants-15-01000-s001.zip › 11Tab. S5 Changes in stomatal density of P. vulgaris varieties at each experimental site.jpg]

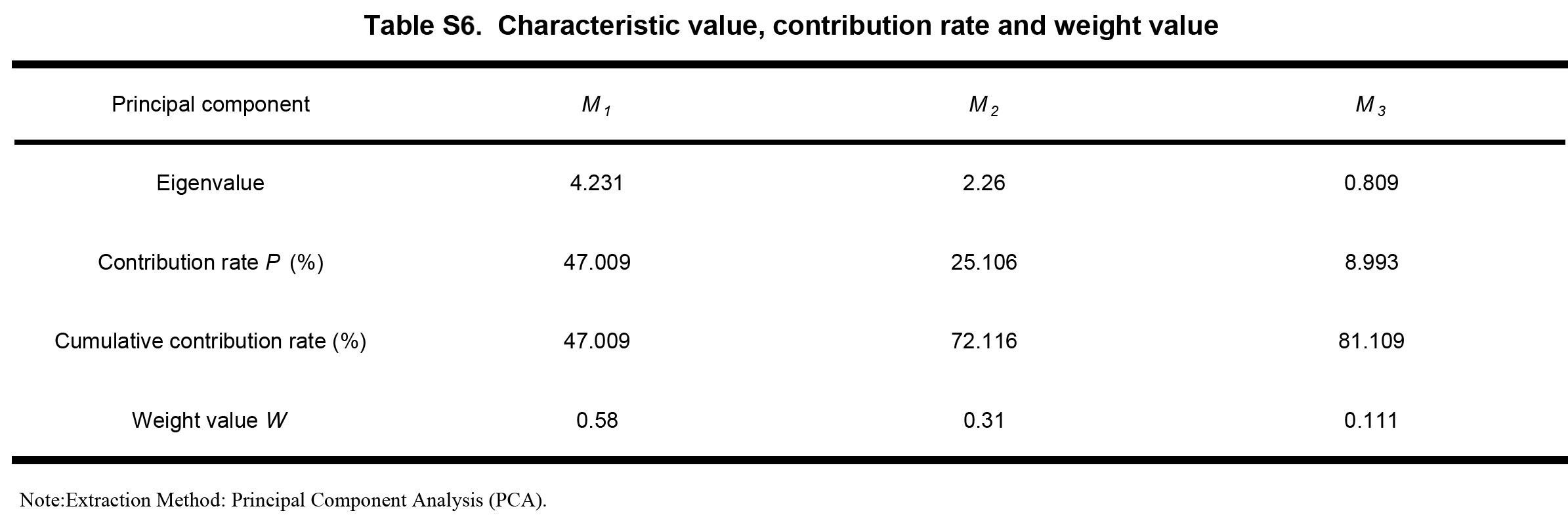

Supplement: Supplementary file 1 [file plants-15-01000-s001.zip › 12Tab. S6 Characteristic value, contribution rate and weight value.jpg]

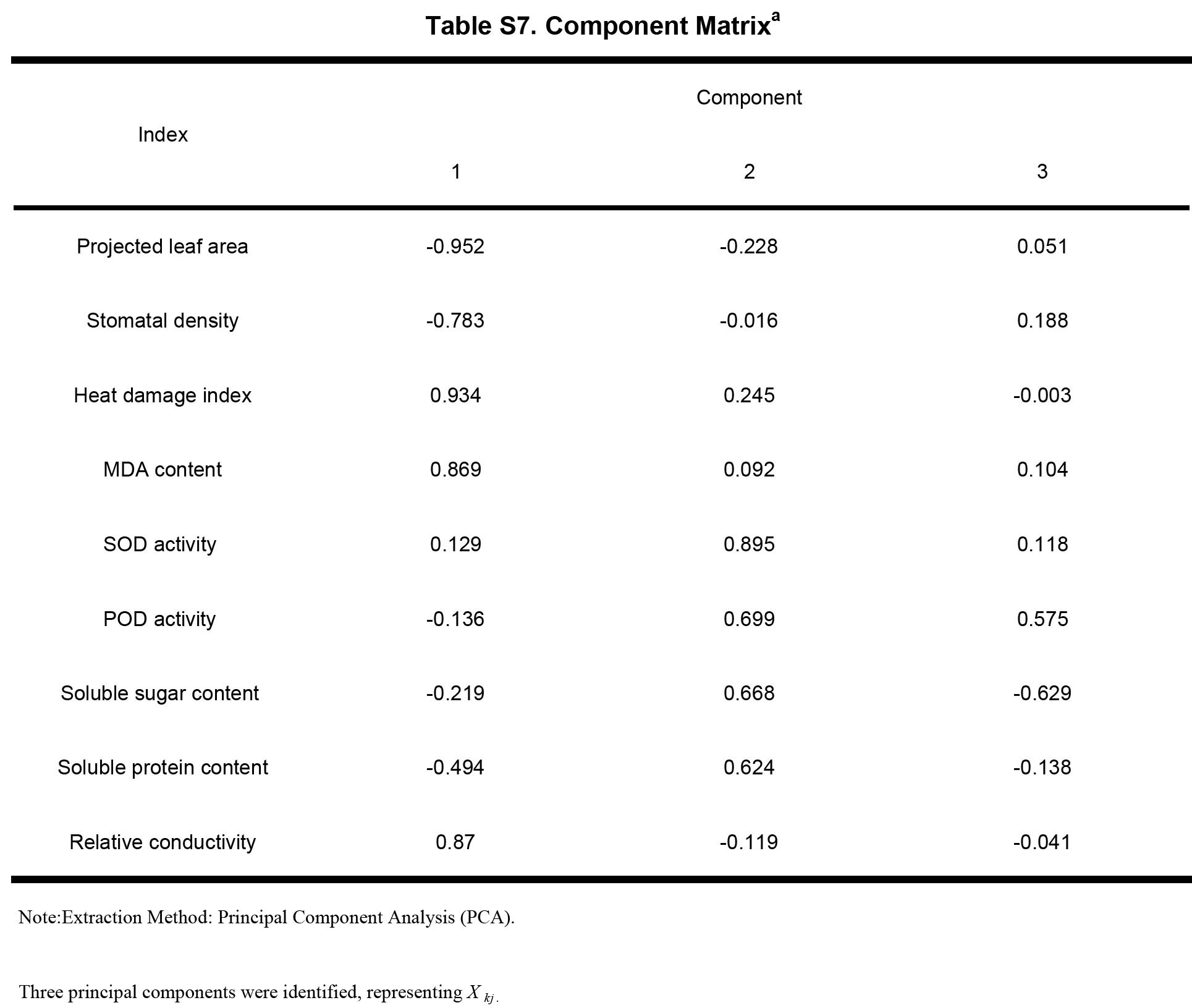

Supplement: Supplementary file 1 [file plants-15-01000-s001.zip › 13Tab. S7 Component Matrixa.jpg]
